# *Cymbopogon proximus* essential oil promotes wound healing and attenuates inflammation through mechanisms supported by network pharmacology and molecular docking

**DOI:** 10.1038/s41598-026-55294-2

**Published:** 2026-06-04

**Authors:** Magdy E. Hanna, Dina S. Ghallab, El Moataz Bellah El Naggar, Mohamed F. Dekinash, Maged W. Helmy, Doaa A. Ghareeb, Eman Shawky

**Affiliations:** 1 Pharmacognosy Department, Faculty of Pharmacy, Damanhour University, Damanhour, El Behira Egypt; 2 Department of Pharmacognosy, Faculty of Pharmacy, Alexandria University, Al Khartoom Square, Alexandria, 21521 Egypt; 3 Pharmacology and Toxicology Department, Faculty of Pharmacy, Damanhour University, Damanhour, El Behira Egypt; 4 Bio‑Screening and Preclinical Trial Lab, Biochemistry Department, Faculty of Science, Alexandria University, Alexandria, Egypt; 5 Center of Excellence for Drug Preclinical Studies (CE-DPS), Pharmaceutical and Fermentation Industry Development Center, City of Scientific Research & Technological Applications (SRTA-City), New Borg El Arab, Alexandria, Egypt; 6 Research Projects Unit, Pharos University, Alexandria, Egypt

**Keywords:** *Cymbopogon proximus*, Ethnopharmacology, Essential oil, Wound healing, Anti-inflammatory activity, GC–MS, Biochemistry, Computational biology and bioinformatics, Drug discovery

## Abstract

*Cymbopogon proximus* Chiov. (Poaceae), widely used in traditional medicine for inflammatory conditions, was investigated for its anti-inflammatory and wound-healing pharmacological potential through an integrated phytochemical, biological, and in silico approach. Essential oils from *Cymbopogon proximus* and *Cymbopogon citratus* were isolated by hydrodistillation and chemically characterized by gas chromatography–mass spectrometry coupled with chemometric discrimination. Biological screening first assessed safety and anti-inflammatory activity in vitro using cytotoxicity evaluation, human red blood cell membrane stabilization, and modulation of inflammatory gene expression in lipopolysaccharide-stimulated white blood cells. Based on the superior in vitro profile, *C. proximus* essential oil was advanced to in vivo pharmacological evaluation in an excisional wound model, with assessment of wound contraction, histopathology, and inflammatory biomarkers. *C. proximus* exhibited a distinct piperitone-dominant volatile profile and demonstrated stronger anti-inflammatory activity than *C. citratus*, evidenced by membrane-stabilizing effects and downregulation of key pro-inflammatory mediators, particularly tumor necrosis factor alpha, within non-cytotoxic concentrations. Topical administration accelerated wound closure, improved re-epithelialization, and reduced inflammatory cell infiltration, with activity comparable to a reference topical preparation. Network pharmacology and molecular docking further supported a multi-target mechanism involving inflammation- and repair-related signaling nodes. Collectively, these findings identify *C. proximus* essential oil as a promising multi-component anti-inflammatory agent with therapeutic potential in inflammation-driven wound repair.

## Introduction

Inflammation is the cornerstone of the innate immune response, serving as the immediate, highly regulated reaction of vascularized tissue to injury, infection, or environmental stressors^1^. This critical process involves the sequential release of pro-inflammatory mediators such as cytokines and the orchestrated infiltration of leukocytes, primarily neutrophils and later macrophages to neutralize threats and clear cellular debris^2^. This inflammatory cascade is most clearly and fundamentally demonstrated in the response to tissue trauma, such as a skin wound.

Skin wounds may arise from extrinsic factors, including surgical incisions, accidental injuries, or traumatic events, as well as from intrinsic factors, such as infections and chronic ulcers^3^. Wound healing is, at its core, a highly regulated instance of inflammation and subsequent resolution. The process of skin wound healing constitutes a complex biological response, integrating cellular, molecular, and humoral components to restore tissue integrity following injury^4,5^.

Current clinical strategies for controlling inflammation and promoting tissue repair often depend on pharmacotherapies such as corticosteroids and non-steroidal anti-inflammatory drugs (NSAIDs), alongside broad-spectrum antibiotics to manage infection^6^. While these synthetic agents are vital in acute care, their long-term or extensive use presents substantial clinical challenges, including severe systemic side effects (e.g., gastrointestinal toxicity and immunosuppression) and, critically, the global threat of antimicrobial resistance^7^. This combination of therapeutic limitations suggests the utility of exploring natural and tolerable alternatives for managing wounds and inflammatory conditions.

Medicinal plants, representing a significant reservoir of bioactive compounds with reported anti-inflammatory and regenerative capabilities, merit investigation in wound mitigation. Genus *Cymbopogon*, belonging to the Poaceae family, comprises approximately 140 species that thrive predominantly in tropical and subtropical regions worldwide. Due to their wide-ranging applications in pharmaceuticals, cosmetics, food, flavouring, and agriculture, these grasses are extensively cultivated, particularly in warmer climates^8^. Among the various aromatic species within this genus, *C. citratus* (West Indian lemongrass), *C. flexuosus* (East Indian lemongrass), and *C. winterianus* (citronella) are the primary sources of commercially valuable essential oils^9,10^. Another species, *C. proximus,* known locally in Egypt as “Halfa bar,” is a common desert plant reputed in traditional Egyptian medicine for its diuretic and antispasmodic benefits on renal conditions^11^.

*C. citratus* is widely cultivated in tropical and subtropical regions of Asia, Africa, and the Americas for its valuable essential oils^12^. The plant typically yields 1–2% essential oil on a dry weight basis, though the composition can vary significantly depending on genetic factors, environmental conditions, and agricultural practices^13^. The essential oil of *C. citratus* is notably rich in citral, comprising the isomers neral and geranial, which together can constitute up to 80% of the oil^14^. Meanwhile, *C. proximus* is found across Central and Northern Sudan, as well as in the Egyptian desert and the sandy coastal regions along Egypt’s southern Red Sea border.

In traditional folk medicine, *C. citratus* leaves are used for the treatment of inflammatory conditions. Scientific validation of these traditional practices has demonstrated that the polyphenolic-rich fractions of the plant possess significant anti-inflammatory and analgesic properties. Specifically, research has shown that these extracts can inhibit nitric oxide production in macrophages and decrease the expression of pro-inflammatory enzymes^15,16^. *C. proximus* has long been recognized in traditional Egyptian medicine for its efficacy as a renal antispasmodic and diuretic agent^17^. Moreover, C. proximus has demonstrated preliminary potential in wound healing. Its rich profile of phytoconstituents— specifically phenolic compounds and flavonoids, is thought to accelerate tissue repair by regulating inflammatory pathways, stimulating collagen production, and facilitating re-epithelialization (Mohamed et al. 2022). Consequently, botanical interventions are increasingly studied in wound care for their biocompatibility, affordability, and minimal adverse effects. Equally important, aligning with literature, *Cymbopogon* species have demonstrated promising anti-inflammatory potential. Specifically, the bioactive fraction of *C. proximus,* characterized by a high concentration of phytochemicals such as flavonoids, coumarins, and stilbenes, demonstrated potent anti-inflammatory activity. This effect was achieved through a significant reduction in the production of key inflammatory cytokines, most notably Tumor Necrosis Factor-alpha (TNF-α)^18^. The bioactive extract of *C. citratus*, which integrates a diverse profile of terpenes, flavonoids, and phenolic compounds, has demonstrated significant anti-inflammatory efficacy across various skin disorders^19^.

*Cymbopogon proximus* Chiov., locally known in Egypt as “Halfa Bar,” holds a long-standing role in traditional Egyptian and Sudanese medicine. Ethnomedical records describe its use as a diuretic, renal antispasmodic, anti-inflammatory agent, and remedy for urinary tract disorders, abdominal pain, and fever^11,17^. Decoctions and infusions prepared from the aerial parts are commonly employed in folk practice, while the aromatic oil has historically been used for topical applications in inflammatory and painful conditions.

As medical research increasingly demands robust evidence, network pharmacology has emerged as a supportive methodology. This technique provides a clear biological framework that helps us understand the pharmacological effect of a drug, not just on one target, but on the entire disease system. It works by establishing a comprehensive map, or a network, connecting the main compounds, the disease targets, and the related pathways^20^. This approach is well-suited for studying the efficacy mechanism of traditional herbal medicines with multiple components that work simultaneously on many targets (multi-component and multi-target effects)^21^.

To address these objectives, our current research aimed to characterize the volatile components of essential oils from two *Cymbopogon* species, *C. citratus* and *C. proximus,* using GC–MS analysis combined with chemometrics, exploring inter-species chemical variability. Then, various in vitro anti-inflammatory tests were preliminarily conducted to evaluate the biologically active *Cymbopogon* species, which was rationally subjected to an excisional wound healing model in mice to examine its potential wound healing through biochemical analysis, histopathology, and epithelialization scores assessment. Complementarily, to objectively gain mechanistic insights beyond the anti-inflammatory and regenerative effects of *C. proximus,* network pharmacology integrated with molecular docking analyses were implemented, providing a comprehensive, system-level view of how the bioactive compounds found in *C. proximus* essential oil affect wound mitigation. This integrative data combining network pharmacology with experimental validation aims to provide a theoretical and experimental basis for further investigating *C. proximus* as a potential therapeutic agent for wound healing; however, further safety studies and extensive clinical trials are warranted.

## Experimental

### Plant material

Leaves of *C. citratus* (https://powo.science.kew.org/taxon/urn:lsid:ipni.org:names:396896-1) and *C. proximus* (https://powo.science.kew.org/taxon/urn:lsid:ipni.org:names:396998-1) (2000 g of each) were collected in November 2024. Plant materials were collected and identified by Dr. Salama Mohamed El-Darier, professor of Plant Ecology Botany and Microbiology Department faculty of Science Alexandria university, Egypt. All experimental research on plants complied with relevant institutional, national, and international guidelines. Necessary permits were obtained for the collection of plant material from the faculty of Science Alexandria university. Two voucher specimens (No.CC-40 for *C. citratus* and CP-41 for *C. proximus*) were kept in the herbarium of Pharmacognosy Department, Faculty of Pharmacy, Damanhour University.

Plant material was collected from three independent biological replicates of each species, where each replicate consisted of leaves harvested from spatially separated plants within the same population. Essential oils were extracted independently from each replicate batch, and GC–MS analysis was performed on all replicate oil samples to ensure analytical reproducibility and biological consistency. Reported volatile compositions represent the mean relative abundance across biological replicates, unless otherwise stated.

### Volatile oils extraction

Parallel with literature^22^, volatile oils were obtained from 250 g of dried leaves from each studied *Cymbopogon* species via hydro-distillation using a Clevenger-type apparatus for 4 h. After separating the oils from the condensed water, they were stored in tightly sealed amber vials at 4 °C. The oil volume was determined using the graduated tube of the apparatus, and the yield was calculated as a percentage relative to the dry weight of the plant material.

### Gas chromatography–mass spectrometry analysis

Volatile component profiling of the oils from the two studied plants was performed using a Trace GC-TSQ mass spectrometer (Thermo Scientific, Austin, TX, USA) equipped with a direct capillary column (TG–5MS; 30 m × 0.25 mm × 0.25 µm film thickness). The oven temperature program began at 45 °C, increased at a rate of 5 °C/min to 250 °C (held for 2 min), and finally reached 300 °C at a rate of 30 °C/min (held for 2 min). The injector and MS transfer line temperatures were set at 270, 260 °C respectively. Helium served as carrier gas at a constant flow rate of 1 mL/min. Following a 2 min solvent delay, 1 µL of each diluted sample was injected automatically using an AS1300 autosampler in split mode. EI mass spectra were collected at 70 eV over an m/z range of 50–650 in full scan mode, with the ion source temperature set at 200 °C. The components were identified by comparison of their mass spectra with those of WILEY 09 and NIST 14 mass spectral databases. Relative retention indices were calculated for each compound, affirming metabolites assignment. Moreover, percentages of the identified volatiles were calculated using GC peak areas without any correction factors and were calculated relatively^23^.

### Multivariate data analysis

Multivariate data analysis of the GC–MS data matrices was performed using SIMCA-P software, version 14.1 (Umetrics, Sweden). Aligning with our rational protocol^24^, techniques such as principal component analysis (PCA), hierarchical cluster analysis (HCA), and orthogonal projections to latent structures-discriminant analysis (OPLS-DA) were employed to reveal clustering patterns among the examined samples based on their volatile constituents.

### Preliminary in vitro anti-inflammatory screening of different Cymbopogon species

#### Assessment of cytotoxicity of different Cymbopogon essential oils by MTT assay

The concentration range selected for in vitro testing was guided by preliminary cytotoxicity screening (MTT assay) to ensure biological relevance while avoiding nonspecific cytotoxic effects. Concentrations were chosen to remain below the EC₁₀₀ threshold, representing doses that preserved normal white blood cell viability. This strategy allowed evaluation of anti-inflammatory activity within a non-toxic pharmacological window, consistent with established essential oil screening practices.

To evaluate the safety profile of the *Cymbopogon* species, cytotoxicity of each essential oil was preliminarily determined on white blood cells using MTT colorimetric assay^25^. White blood cells (WBCs) were seeded in 96-well plates at a density of 1 × 10^5^ cells/well in 200 (μL) of RPMI medium. The cells were treated with a range of *Cymbopogon* essential oil concentrations and incubated for 72 h at 37 °C under a humidified atmosphere of 5% CO2. Post-incubation, 20 μL of MTT solution (5 mg/mL in PBS, pH 7.4) was added to each well and incubated for an additional 4 h. This process facilitates the reduction of MTT by viable cell mitochondrial dehydrogenases into water-insoluble violet formazan crystals. These crystals were subsequently solubilized in 100 μL of dimethyl sulfoxide (DMSO). The optical density (OD) was measured using a spectrophotometer at a wavelength of 570 nm. Statistical analysis and curve fitting were conducted using GraphPad InStat software to determine the Effective Concentration 100% (EC100), defined as the maximum concentration maintaining 100% cellular viability. The percentage of viable cells was calculated using the following equation:

The following equation could be applied to determine the percentage of the viable cells:

$$\% {\text{ cells viable }} = \, \left( {{\mathrm{AT}} - {\text{Ab }}/{\mathrm{AC}} - {\mathrm{Ab}}} \right){\text{ x 1}}00$$

AT = mean absorbances of the treated cells with different concentration levels of each *Cymbopogon* essential oil.

Ab = mean absorbances of cells treated with vehicle including (RPMI) deprived from fetal bovine serum.

AC = mean absorbances of control untreated cells exposed to growth medium only.

#### In vitro* anti-inflammatory testing of different Cymbopogon species using human red blood cell membrane stabilizing method*

All methods were carried out in accordance with relevant guidelines and regulations, including the NIH guidelines for research involving human subjects. All experimental protocols were approved by Damanhour Faculty of Pharmacy Research Ethics Committee. Informed consent was obtained from all subjects for the collection of samples. The anti-inflammatory potential of the plant essential oils was evaluated using the human red blood cell (HRBC) membrane stabilization assay. This model is biologically relevant because the HRBC membrane serves as an analog for the lysosomal membrane. In the inflammatory response, the rupture of lysosomal membranes leads to the extracellular release of hydrolytic enzymes and proteases, which induce tissue damage and exacerbate various inflammatory disorders. By inhibiting hypotonicity-induced membrane lysis, the essential oils demonstrate their ability to stabilize membrane integrity, thereby mimicking a key anti-inflammatory mechanism. This assay was conducted in accordance with the protocol established by (Sadique et al. 1989).

#### 2.5.3. In vitro* anti-inflammatory screening of different Cymbopogon species through inflammatory* gene expression monitoring in LPS-stimulated white blood cells

To establish an in vitro model for acute inflammation, human white blood cells (WBCs) were stimulated by adding 50 μl of Lipopolysaccharide (LPS) derived from *Escherichia coli* to CO2 incubator, the plate was centrifuged at 1650 rpm for 5 min, and the supernatants containing secreted inflammatory factors were carefully discarded. Subsequently, 200 µl of *Cymbopogon* essential oils at various concentration levels were introduced, along with the reference anti-inflammatory drug Piroxicam. The plate was then incubated for an additional 3 days. The Effective Anti-inflammatory Concentrations (EAICs) were subsequently calculated. This metric serves as a critical measure of potency, where a lower EAIC value signifies superior anti-inflammatory potential. To gain mechanistic insight, quantitative real-time reverse transcription PCR (RT-qPCR) was utilized to quantify the expression levels of genes encoding key pro-inflammatory mediators, specifically TNF-α, IL6, IL-1β and INF-γ. The final gene expression results were reported as mean values ± standard deviation (SD) of three individual replicates. Furthermore, the overall cellular response magnitude to the anti-inflammatory treatment was monitored and quantitatively assessed using the Stimulation Index (SI). The SI serves as a crucial quantitative score in the anti-inflammatory evaluation, comparing the cellular response induced by the treatment or stimulus relative to that of an untreated control group. This index was calculated according to the formula:

$$\begin{gathered} {\text{Stimulation index }}\left( {{\mathrm{SI}}} \right) \, = \hfill \\ \frac{{{\text{Mean absorbance of LPS}} - {\text{stimulated untreated cells or LPS}} - {\text{stimulated cells treated with the }}Cymbopogon{\text{ extracts}}}}{{\text{Mean absorbance of control untreated cells containing cell culture growth medium only}}} \hfill \\ \end{gathered}$$

RT-qPCR) was employed to determine the expression levels of the genes encoding key pro-inflammatory mediators, including TNF-α, IL-1β, IFN, and IL-6. To prepare for subsequent gene expression analysis, the cellular pellets were subjected to an RNA isolation protocol based on the spin column methodology. Initially, the pellets were lysed by combining them with 50 µl of Solution R1 for 30 s, followed by a 1-min incubation at ambient temperature. Subsequently, 300 µL of Solution R2 was added to facilitate binding, and the mixture was centrifuged for 3–5 min at 4 °C. The resulting supernatant, containing the target RNA, was loaded onto a spin column and centrifuged at 14,000 rpm at 4 ºC for 30 s. The flow-through was discarded at this stage. Purification was achieved by introducing 300 µl of phosphate buffer to the spin column, followed by centrifugation at 10,000 rpm for 1 min, a step repeated twice to ensure optimal wash. Finally, the RNA was eluted into a sterile 1.5 ml microcentrifuge tube using 30 µl of elution buffer. This final mixture was incubated at room temperature for 1 min and then centrifuged for 30 s at 14,000 rpm at 4 ºC. The quality and concentration of the isolated RNA were assessed spectrophotometrically. Optical Density (OD) readings were taken, revealing the absorbance and purity at A260/A280 nm, respectively and preserved at -80 °C until real-time (RT-qPCR). For cDNA synthesis, 2 µg of the total RNA was mixed with oligo deoxythymidine bases (dT) and nuclease-free water. The mixture was heat-denatured at 65ºC for 5 min before immediate cooling on ice. Subsequent mixing with the reaction buffer 5X, dNTPs mix, RNase inhibitor, and reverse transcriptase allowed for complementary DNA (cDNA) synthesis through incubation at 42ºC for 1 h, followed by enzyme inactivation at 70ºC for 5 min. Gene expression levels for the target inflammatory markers were assessed using the synthesized cDNA and specific gene primers (Table 1) combined with 2 X SYBR green master mix. A non-template control (NTC) was included to exclude reagent contamination or primer dimers. The PCR cycling program involved initial denaturation (1 cycle of 95ºC for 10 min), followed by 40 cycles of denaturation (95ºC for 15 s), annealing (at 60ºC for 30 s) and extension (at 72 ºC for 30 s).

**Table 1:** The inflammatory gene-specific primers utilized in RT-qPCR assay.

Gene	Primer
TNF-α	F-CTCTTCTGCCTGCTGCACTTTG
	R- ATGGGCTACAGGCTTGTCACTC
IL-6	F, 5′-TGAACTCCTTCTCCACAAGCG-3′
	R, 5′-TCTGAAGAGGTGAGTGGCTGTC-3′
IL-1β	F, CCACAGACCTTCCAGGAGAATG
	R, GTGCAGTTCAGTGATCGTACAGG
INF-γ	F, GAGTGTGGAGACCATCAAGGAAG
	R, TGCTTTGCGTTGGACATTCAAGTC
GAPDH	F, GGATTTGGTCGTATTGGG
	R, GGAAGATGGTGATGGGATT

The fold change in gene expression, reflecting the effect of LPS and the *Cymbopogon* essential oils on gene expression, was calculated using the comparative threshold cycle

$$\Delta {\text{CT normal }} = {\text{CT normal untreated cells }}{-}{\text{ CT reference}}$$

$$\Delta {\text{CT induced }} = - {\text{ CT LPS}} - {\text{stimulated cells }}{-}{\text{ CT reference}}$$

$$\Delta {\text{CT tested plant essential oil }} = {\text{ CT tested plant essential oil}} - {\text{treated cells }}{-}{\text{ CT reference}}$$

In case of genes:

$$\Delta \Delta {\text{CT tested plant essential oil }} = \, \Delta {\text{CT tested plant essential oil}}{-} \, \Delta {\text{CT normal}}$$

$$\Delta \Delta {\text{CT induced }} = \, \Delta {\text{CT induced }}{-} \, \Delta {\text{CT normal}}$$

$${\text{Fold change in gene expression }} = { 2}^{{ - \Delta \Delta {\mathrm{CT}}}}$$

where:

CT normal: threshold cycle value of genes of extracted mRNA of untreated control cells.

CT reference: threshold cycle value of GAPDH which is used as a housekeeping gene or internal control.

CT induced: threshold cycle value of gene of extracted mRNA of LPS-stimulated white blood cells.

CT tested *Cymbopogon* essential oil: threshold cycle value of genes of extracted mRNA of *Cymbopogon* essential oil treated-LPS-stimulated white blood cells which is known as the cycle number at which the fluorescence generated within a reaction crosses the fluorescence threshold.

### In vivo* evaluation of the wound healing potential of C. proximus in a mouse excision model*

To select an appropriate topical dose of *C. proximus* essential oil (EO) for the main efficacy experiment, we conducted a preliminary dose-finding pilot. Three dose levels (low, medium, high) were tested in separate groups (*n* = 3/group). The EO formulation was applied topically once daily at 5% (low), 10% (medium), and 20% (high). Screening endpoints were (i) wound area reduction and contraction rate and (ii) inflammatory biomarkers (TNF-α, IL-2, TGF-β1). The selected dose used in the main study was chosen based on the best balance of wound closure performance and biomarker modulation without local tolerability concerns.

In our rationally established workflow, since *C. proximus* revealed promising anti-inflammatory results in preliminary in vitro analyses, it was accordingly subjected to an excisional wound healing model. All animal procedures and experimental protocols were conducted in accordance with the relevant guidelines and regulations (AVMA Guidelines) and were approved by Damanhour Faculty of Pharmacy Research Ethics Committee (Approval No. 425PG18, 30 April 2025). The experimental protocol was strictly conducted in accordance with the guidelines for the Care and Use of Laboratory Animals. Mice were sourced from the Animal House of the Medical Research Institute, Alexandria University, Egypt. The mice were acclimated for one week under standard laboratory conditions.

At the conclusion of the experimental period, all mice were humanely euthanized to allow for the collection of skin tissue samples for histological and phytochemical evaluation. Euthanasia was performed using a chemical overdose of Thiopental Sodium at a concentration of 150 mg/kg. The agent was administered via the intraperitoneal route using sterile 1-mL syringes. This method was selected to ensure a rapid and painless transition to unconsciousness followed by cardiac arrest. Death was confirmed by the permanent cessation of the heartbeat and the absence of the pedal withdrawal reflex, ensuring all procedures complied with international humane endpoint standards. This research did not involve human participants, human data, or human tissue samples.

In traditional folk practice, aromatic plant oils and decoctions are typically applied topically in diluted form to inflamed or injured skin. Accordingly, the experimental topical concentration used in this study was designed to approximate culturally relevant dilution practices^26^, while remaining within experimentally safe and pharmacologically active limits.

The topical concentration of *C. proximus* essential oil (10% v/w) was selected based on three criteria:

1. Safety margins derived from in vitro cytotoxicity screening,
2. Concentrations commonly reported in topical essential oil wound-healing formulations in experimental and traditional medicine contexts, and.
3. The need to ensure sufficient local bioavailability at the wound surface without inducing dermal irritation or systemic toxicity.

This concentration falls within the range previously reported for safe topical application of monoterpene-rich essential oils in murine wound models.

For the in vivo assessment, the experimental animals (*n* = 24) were randomly allocated into four distinct groups (*n* = 6 per group, consisting of an equal distribution of males and females): model Group (Wound), positive control group (Mebo®), negative control group (receiving the base carrier-vehicle-), and the treated group (*C. proximus* essential oil). Removing a fragment from the skin using a 12 mm diameter circular biopsy punch^27^. Measuring the wound area immediately using a digital caliper and repeating the procedure on Days 4, 7, 11 and 14 after wound creation to assess the wound healing progress. Before the induction of the excisional wound, all mice were anesthetized to ensure a complete lack of pain perception and to maintain surgical immobility. Anesthesia was achieved via intraperitoneal injection of Thiopental Sodium at a dose of 50 mg/kg body weight. The depth of anesthesia was monitored throughout the procedure by the loss of the pedal withdrawal reflex. This dose provided approximately 20–30 min of surgical anesthesia, allowing for the precise creation of the wound model under aseptic conditions. Following the procedure, mice were placed in a warm, quiet recovery cage and monitored until they regained full consciousness and normal locomotor activity.

The *C. proximus* essential oil (10%) was administered to the assigned groups for a period of 14 days. The selection of this concentration is supported by recent literature. For instance, research on *Cymbopogon* herbal formulations has demonstrated that a 10% concentration provides an optimal balance between therapeutic efficacy and preparation stability, showing superior wound-healing results compared to lower doses without the localized irritation often associated with higher concentrations^26^. At the end of the experimental duration, animals were euthanized and skin tissues samples, at the area of excisional wound were collected, processed for histological evaluation using Hematoxylin and Eosin (H&E) staining, and analysed for inflammatory biomarkers (IL-2, TGF-β1, and TNF-α) using ELISA kits: IL-2 (Catalog No. E-EL-M0042, United States), TGF-β1 (Catalog No. E-EL-M0051, United States), and TNF-α (Catalog No. CSB-E04741m, China). The data obtained from the analysis of stained tissue sections, as well as from the samples assessed for inflammatory biomarkers, were subjected to statistical evaluation. A one-way ANOVA was performed using GraphPad Prism 8 software to determine the significance of differences among the experimental groups.

#### Histological evaluation of wounds

The qualitative histology studies were performed by analysing the stained tissues using a specific score for each parameter, according to described below:

Inflammation degree was evaluated via monitoring the cellular infiltration (polymorphonuclear and mononuclear) in HE-stained sections using the 10 × objective. Further, evaluating the amount of scab formed in the wound tissue by HE-stained sections analysis using the 4 × objective. Relatedly, Epithelialization index in HE-stained sections was assigned using the 10 × objective. All these scores stated above were listed in Table 2**.**

**Table 2:** Morphological scores of inflammations, scab, and epithelialization in H&E-stained histological sections of excisional Wistar rats’ wounds.

Score	Inflammation parameter	Scab parameter	Epithelialization parameter
0	WHOLE SKIN–absence of inflammation	ABSENCE	WHOLE SKIN–whole epithelium
1	DISCRETE presence of few inflammatory cells	DISCRETE	DISCRETE–partial epithelization with a small new epithelial layer (the epithelial tongue occupies, at most, 1/3 of the wound gap)
2	MODERATE–many inflammatory cells	MODERATE	MODERATE–partial epithelization with a longer new epithelial layer (the epithelial tongue occupies more than 1/3 of the wound gap)
3	SEVERE–exaggerated inflammatory cellularity	SEVERE	Complete EPITHELIZATION

### Network pharmacology study

To gain mechanistic insights beyond the efficacy of *C. proximus* essential oil components in wound healing, the characterized compounds gained from GC–MS analysis of *C. proximus* essential oil were designated as the candidate compounds for network pharmacology analysis.

Accordingly, the target gene analysis of the annotated compounds was conducted using referenced genomic databases, namely STITCH (http://stitch.embl.de/, ver. 5.0) and Swiss Target Prediction (http://www.swisstargetprediction.ch/), with the species designated as “*Homo sapiens*.” Coincidently, the main target genes relevant to “wound” were pinpointed from the Therapeutic Target Database (TTD) and Gene Cards databases. Then, to find the shared targets between wound and *C. proximus* essential oil compounds, a Venn diagram was created using the Venny 2.0 platform (https://bioinfogp.cnb.csic.es/tools/venny/index2.0.2.html). The targets allocated in the intersection were submitted to the STRING database (https://string-db.org/) to establish a protein–protein interaction (PPI) network based on the functional associations among the target proteins. The UniProt database (http://www.uniprot.org/) was used as a standardized, high-quality tool for target gene identification.

Next, the DAVID Bioinformatics platform (https://david.ncifcrf.gov) was utilized to profile the functional pathways relating to the hub genes through the Kyoto Encyclopedia of Genes and Genomes (KEGG) enrichment analysis, where items with a cutoff of False Discovery Rate (FDR) ≤ 0.05 were considered statistically significant. Relatedly, a bubble chart of KEGG analysis items visualizing the top 15 significantly enriched biological pathways was built. Of note, all the interactive networks of components, genes, and pathways were established and analyzed using Cytoscape 3.7.1 (Cytoscape Consortium, CA, USA), where degree values, betweenness centrality, and closeness centrality provided in a network analysis plug-in were used to evaluate the importance of nodes in each network^28,29^.

To provide an intuitive, quantitative overview of the many-to-many relationships between the main biological components (compounds, targets, and pathways) in the network pharmacology analysis, a Sankey diagram was created using the bioinformatics platform (https://www.bioinformatics.com.cn/en), summarizing the main efficacy mechanisms of *C. proximus* essential oil components in controlling the inflammatory responses and protecting cells from damage, thereby facilitating the natural healing process.

### Molecular docking analysis

Molecular docking is a cornerstone of modern drug discovery and structural biology, serving as an in silico method to predict how a small molecule (ligand) interacts with a macromolecular target like a protein^30,31^.

With a scope to complement the network pharmacology findings, molecular docking analysis was implemented to prioritize the binding poses (orientations) and affinities between the top-scoring *C. proximus* compounds and the hub genes using Schrodinger Maestro 11.8 software (LLC, New York, NY). Practically speaking, the X-ray crystal structures of Mitogen-activated protein kinase 1 (MAPK1), Prostaglandin G/H synthase 2 (PTGS2), and Interleukin-1β (IL-1β) with Protein Data Bank (PDB) entry codes; 4QTB, 5IKR, and 8C3U, respectively were downloaded (PDB format) from the Protein Data Bank (PDB) platform (http://www.rcsb.org/pdb). Aligning with our previous protocol^24,32^, Protein Preparation Wizard module within the Schrödinger software package was utilized to prepare and refine the geometry of the protein structures through assigning bond orders, optimizing hydrogen bond networks, and minimizing the energy. Further, the top candidate compounds were prepared using the LipPrep module to tune the chirality, ring conformations, stereochemistry, and ionization states. Grid Preparation Tool was used to define the binding site coordinates of ≤ 20 Å. Molecular docking analysis was conducted using the Glide 11.8 module, where the GlideScore (G score) was assigned as an empirical scoring function to predict the binding affinity between a ligand and a protein receptor. A more negative G score indicates a lower binding energy and, therefore, a stronger predicted binding affinity and a more stable ligand-receptor complex.

#### Statistical analysis

All experiments were carried out in triplicate, and the results were expressed as mean values and standard deviations. The Statistical difference between samples was determined by one-way ANOVA followed Tukey’s test. *P* < 0.05 was statistically significant.

## Results and discussion

### The variability in the volatile component profiles of two oils studied using GC/MS analysis

To study the inter-specific variability of the volatile components of *C. citratus *and *C. proximus*, volatile oils of the two studied plants (leaves) were collected in November 2024 and analyzed by GC–MS for the identification and quantification of oil components. Table 3 listed forty-three components identified in the volatile oils of the two studied plants. *C. citratus* had a low oil yield percentage of 0.6%, while *C. proximus* yielded a higher oil percentage of 1.4%. The percentage of total identified compounds was 93.62% for the essential oil of *C. citratus*, compared to 85.56% for the oil of *C. proximus*. GC–MS analysis across replicate oil samples demonstrated high compositional consistency, indicating acceptable analytical reproducibility under the applied experimental conditions.

**Table 3:** Chemical composition, relative retention times (RT), and relative percentages (%) of volatile constituents identified in the leaf essential oils of *C. citratus* and *C. proximus *via GC–MS analysis.

	*C. citratus*			*C. proximus*			
Compound Name	RT	Area %	Peak Area	RT	Area %	Peak Area	M. Formula
5-Hepten-2-one, 6-methyl-	5.64	0.48	15,628,942.52	….	….	….	C₈H₁₄O
2,3-Dehydro-1,8-cineole	….	….	….	5.86	0.17	5,355,759.57	C₁₀H₁₆O
Monoterpenes hydrocarbon							
α-Pinene	….	….	….	7.09	0.15	4,615,573.23	C₁₀H₁₆
Camphene	….	….	….	5.06	0.06	1,738,829.53	C₁₀H₁₆
β-Pinene	6.01	10.28	331,916,437.00	….	….	….	C₁₀H₁₆
( +)-2-Carene	….	….	….	6.23	12.25	375,579,379.88	C₁₀H₁₆
D-Limonene	….	….	….	6.88	2.24	68,706,857.89	C₁₀H₁₆
ɑ-Myrcene	7.12	0.13	4,222,838.58	….	….	….	C₁₀H₁₆
(Z)-β-Ocimene	….	….	….	10.31	0.26	7,897,556.25	C₁₀H₁₆
Total monoterpenes hydrocarbon		10.41			14.96		
Oxygenated monoterpenes							
( +)-Fenchone	….	….	….	8.04	0.16	4,853,886.53	C₁₀H₁₆O
1,8-Cineole (Eucalyptol)	8.26	0.01	350,494.49	9.18	0.81	24,946,234.08	C₁₀H₁₈O
Linalool	8.67	0.89	28,367,000.56	….	….	….	C₁₀H₁₈O
p-Menth-2-en-1-ol	….	….	….	9.64	0.55	16,801,748.01	C₁₀H₁₈O
cis-p-Mentha-2,8-dien-1-ol	….	….	….	10.8	0.34	10,301,456.50	C₁₀H₁₆O
Geranyl acetate	16.16	3.03	97,946,046.65	….	….	….	C₁₂H₂₀O₂
Limonene oxide	9.67	0.48	15,648,163.85	….	….	….	C₁₀H₁₆O
trans-Verbenol	10.2	2.18	70,320,443.45	….	….	….	C₁₀H₁₆O
Citronellal	9.9	0.24	7,755,370.09	….	….	….	C₁₀H₁₈O
cis-Verbenol	10.68	3.51	113,512,272.91	….	….	….	C₁₀H₁₆O
α-Terpineol	….	….	….	11.02	1.14	34,956,147.50	C₁₀H₁₈O
Isoneral (β-Citral (neral)-cis)	12.24	31.98	1,033,215,763.00	….	….	….	C₁₀H₁₆O
Piperitone	….	….	….	12.51	53.04	1,626,587,523.12	C₁₀H₁₆O
Geraniol	12.89	4.40	142,105,166.46	….	….	….	C₁₀H₁₈O
Isogeranial(ɑ-Citral (geranial)-trans)	13.05	35.76	1,155,128,009.28	….	….	….	C₁₀H₁₆O
Total oxygenated monoterpenes		81.59			56.04		
Aromatic monoterpenes							
o-Cymene	….	….	….	6.67	0.33	9,971,425.26	C₁₀H₁₄
Sesquiterpenes hydrocarbon							
β-Elemene	….	….	….	16.66	0.25	7,543,375.44	C₁₅H₂₄
Caryophyllene	17.32	0.21	6,913,206.74	17.31	0.24	7,428,717.68	C₁₅H₂₄
β-Bourbobene				17.65	0.09	2,745,653.81	C₁₅H₂₄
ɑ-Bergamotene	17.81	0.18	5,721,933.33	….	….	….	C₁₅H₂₄
γ-cadinene	18.14	0.03	985,851.19	….	….	….	C₁₅H₂₄
delta-cadinene	18.29	0.11	3,645,505.18	….	….	….	C₁₅H₂₄
ɑ-copaene	18.81	0.09	2,755,428.66	….	….	….	C₁₅H₂₄
Epi-α-selinene	….	….	….	19.57	0.18	5,665,888.55	C₁₅H₂₄
α-Selinene	….	….	….	19.79	0.24	7,411,092.02	C₁₅H₂₄
Total Sesquiterpenes hydrocarbon		0.41			0.96		
Oxygenated Sesquiterpenes							
elemol	19.17	0.17	5,563,650.01	20.31	5.14	157,638,167.31	C₁₅H₂₆O
Caryophyllene Oxide	20.98	0.12	3,803,388.39	20.98	0.47	14,401,206.15	C₁₅H₂₄O
ɑ-Cadinol	21.83	0.31	10,045,460.34	….	….	….	C₁₅H₂₆O
α-acorenol	….	….	….	21.88	0.06	1,828,234.93	C₁₅H₂₆O
γ-eudesmol	….	….	….	22.19	1.48	45,535,606.03	C₁₅H₂₆O
γ-Cadinol	22.37	0.03	875,355.00	22.39	0.58	17,781,247.45	C₁₅H₂₆O
β-eudesmol	22.67	0.10	3,184,110.71	22.54	2.34	71,810,797.43	C₁₅H₂₆O
α-eudesmol	….	….	….	22.66	2.92	89,433,939.04	C₁₅H₂₆O
Cubenol	….	….	….	22.92	0.58	17,787,796.10	C₁₅H₂₆O
Total Oxygenated Sesquiterpenes		0.73			13.1		
Total identified cpds		93.620			85.56		
Extraction yield		2 ml/300gm			2.8/200gm		

The main chemical classes of the identified compounds in the two studied plants were monoterpene hydrocarbons, oxygenated hydrocarbons (ethers, ketones, aldehydes, alcohols, and esters), aromatic monoterpenes, sesquiterpene hydrocarbons, and oxygenated sesquiterpenes (alcohols). As shown in Table 3, the most encountered class among the two studied oils was oxygenated monoterpenes, with percentages of 81.59% and 56% in the essential oils of *C. citratus* and *C. proximus*, respectively. Meanwhile, a higher percentage of monoterpene hydrocarbons was recorded in the volatile oil of *C. proximus* (14.95%), while its percentage was 10.41% in *C. citratus*. The lowest percentage of identified classes was for aromatic monoterpenes (0.33%) in the essential oil of *C. proximus*.

Among the studied oils, *C. proximus* oil showed a higher percentage of sesquiterpene hydrocarbons (0.96%) and oxygenated sesquiterpenes (13.1%). Meanwhile, the predominant components of the oil of *C. proximus* were piperitone (monoterpene cyclic ketone, 53.04%), 2-carene (monoterpene hydrocarbon, 12.25%), elemol (sesquiterpene alcohol, 5.14%), α-eudesmol (sesquiterpene alcohol, 2.92%), β-eudesmol (sesquiterpene alcohol, 2.34%), D-limonene (monoterpene hydrocarbon, 2.24%), γ-eudesmol (sesquiterpene alcohol, 1.84%), α-terpineol (monoterpene alcohol, 1.14%), and eucalyptol (monoterpene ether, 0.81%). A previous study interested in the physiochemical investigation of essential oils of *Cymbopogon* species cultivated in Sudan highlighted that the main compounds found in the oil of *C. proximus* were piperitone (43.2%), elemol (13.45%), 2-carene (8.08%), β-eudesmol (5.41%), limonene (2.45%), and α-eudesmol (2.61%) (Elhassan et al., n.d.).

**Fig. 1:**
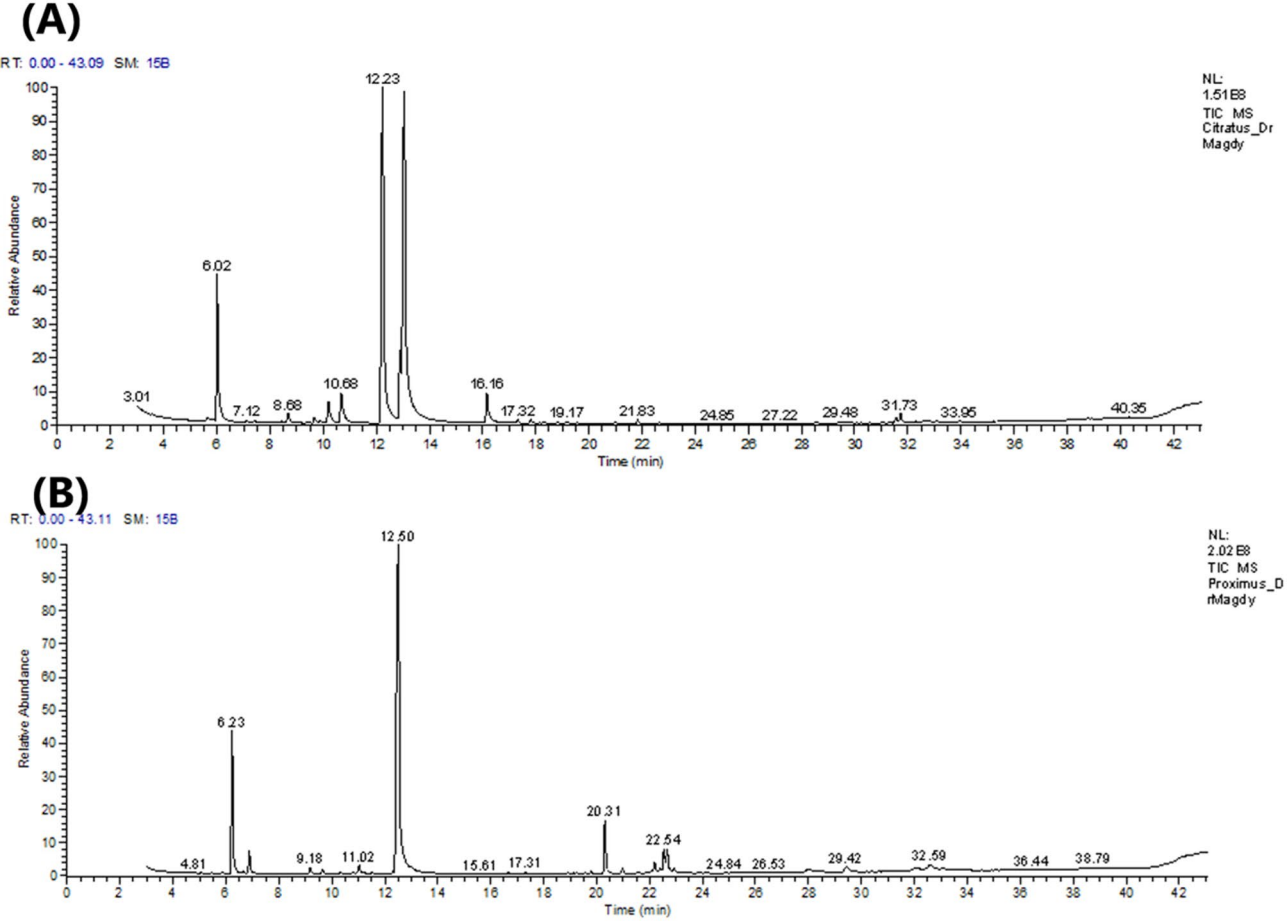
Representative GC-MS total ion chromatograms (TIC) of *C. citratus* (**A**) and *C. proximus* (**B**) essential oils, displaying the main components separated by retention time.

Conversely, *C. citratus* oil contained lower percentages of sesquiterpene hydrocarbons (0.41%) and oxygenated sesquiterpenes (0.73%). The predominant components were the monoterpene aldehydes ɑ-citral (35.76%) and β-citral (31.98%), identified as the most prevalent oxygenated monoterpenes in the oil. Additionally, β-pinene (a monoterpene hydrocarbon, 10.28%), geraniol (a monoterpene alcohol, 4.4%), cis-verbenol (a monoterpene alcohol, 3.51%), geranyl acetate (a monoterpene ester, 3.03%), trans-verbenol (a monoterpene alcohol, 2.18%), linalool (a monoterpene alcohol, 0.89%), and citronellal (a monoterpene aldehyde, 0.24%) were detected in the *C. citratus* oil. A previous study on the volatile components of *Cymbopogon* essential oils (Loko et al., n.d.) showed that the predominant compounds in *C. citratus* were β-pinene (21.9%), ɑ-citral (23.46%), and β-citral (24.64%). These results indicate that the two oils exhibit distinct volatile profiles, highlighting the impact of inter-specific variability on the composition of the tested oils (Fig. 1).

### Multivariate analysis of GC–MS data

**Fig. 2:**
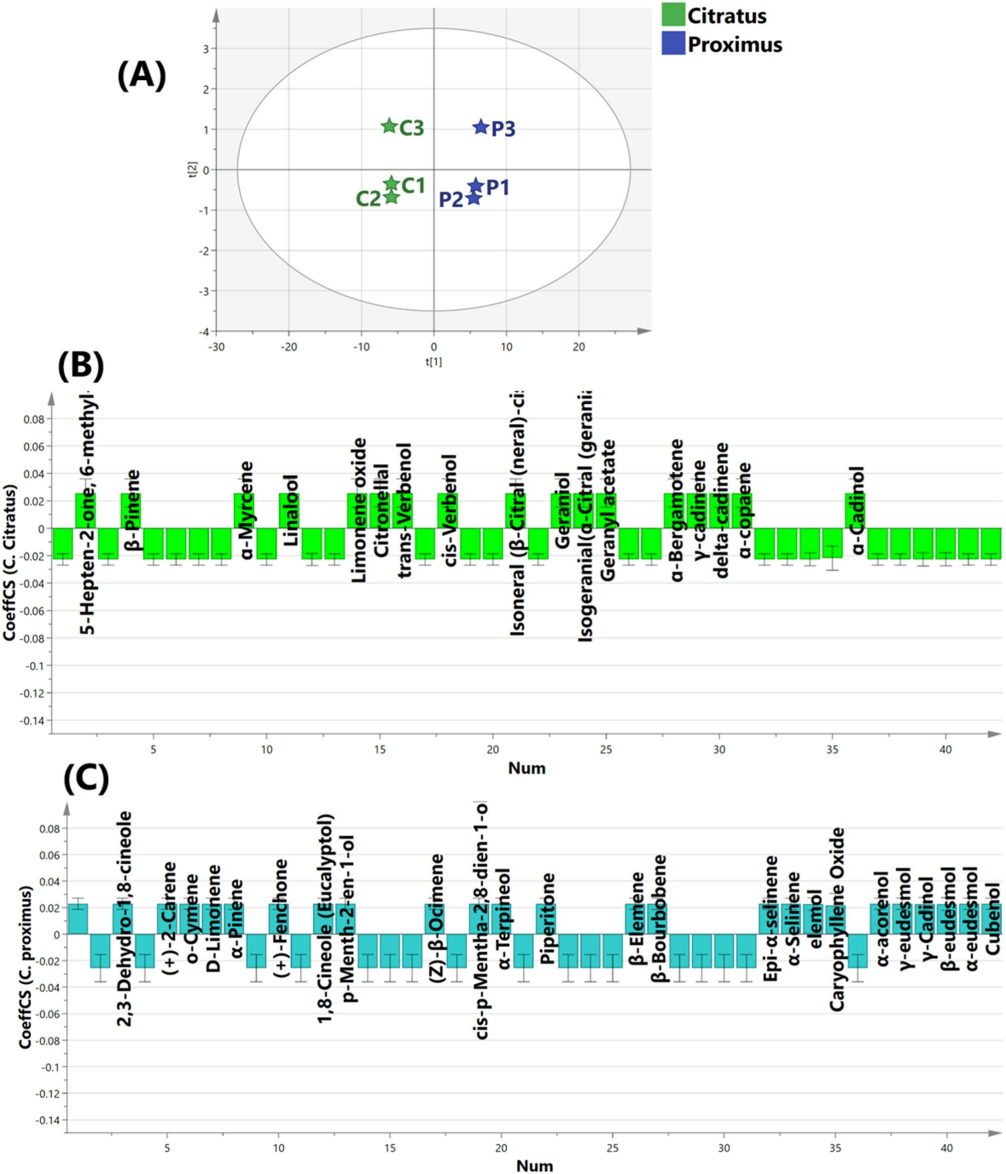
(**A**) Orthogonal Partial Least Squares Discriminant Analysis (OPLS-DA) Score Plot the oil samples of *C. citratus* (green colour) and *C. proximus* (blue colour). (**B**) Coefficient plot of OPLS model to the chemistry of *C. citratus* (green colour) and (**C**) Coefficient plot of OPLS model to the chemistry of *C. proximus* (blue colour).

Principal Component Analysis (PCA) was conducted to evaluate the variation and clustering pattern among the oil samples of *C. citratus* and *C. proximus.* The PCA score plot (Fig. S1) demonstrates a clear separation between the two species along the first principal component where the *C. citratus* samples were clustered together on the left side of the plot, while the *C. proximus* samples were grouped separately on the right side, indicating distinct chemical profiles between the two species. To provide deeper insights into the differences among the samples and revealed their discriminatory markers, OPLS-DA model was conducted. OPLS-DA score scatter plot (Fig. 2A) showed the in between class discrimination of *C. citratus* oil from *C. proximus* oil samples along latent variable 1.

The Coefficient plot of OPLS-DA model Fig. 2B &C displays the discriminatory volatile compounds in the studied oils. For clarity, the volatile compounds which significantly contribute to the chemical profile of *C. citratus* included methyl-5-hepten-2-one, β-pinene, α-myrcene, linalool, limonene oxide, citronellal, trans-verbenol, cis-verbenol, isoneral, geraniol, isogeraniol, geranyl acetate, ɑ-bergamotene, γ-cadinene, delta-cadinene, ɑ-copaene, and α-cadinol. On the other hand, the significant volatile compounds of *C .proximus* were camphene, 2,3-dehydro-1,8-cineole, 2-carene, o-cymene, D-limonene, α-pinene, fenchone, eucalyptol, p-menth-2-en-1-ol, β-ocimene, cis-p-Mentha-2,8-dien-1-ol, α-terpineol, piperitone, β-elemene, β-bourbobene, epi-α-selinene, α-selinene, elemol, caryophyllene, α-acorenol, γ-cadinol, ɑ-eudesmol, β-eudesmol, γ-eudesmol, and cubenol.

### *Preliminary *in vitro* anti-inflammatory screening of different Cymbopogon species*

#### In vitro* anti-inflammatory testing of different Cymbopogon species using human red blood membrane stabilizing method*

**Fig. 3:**
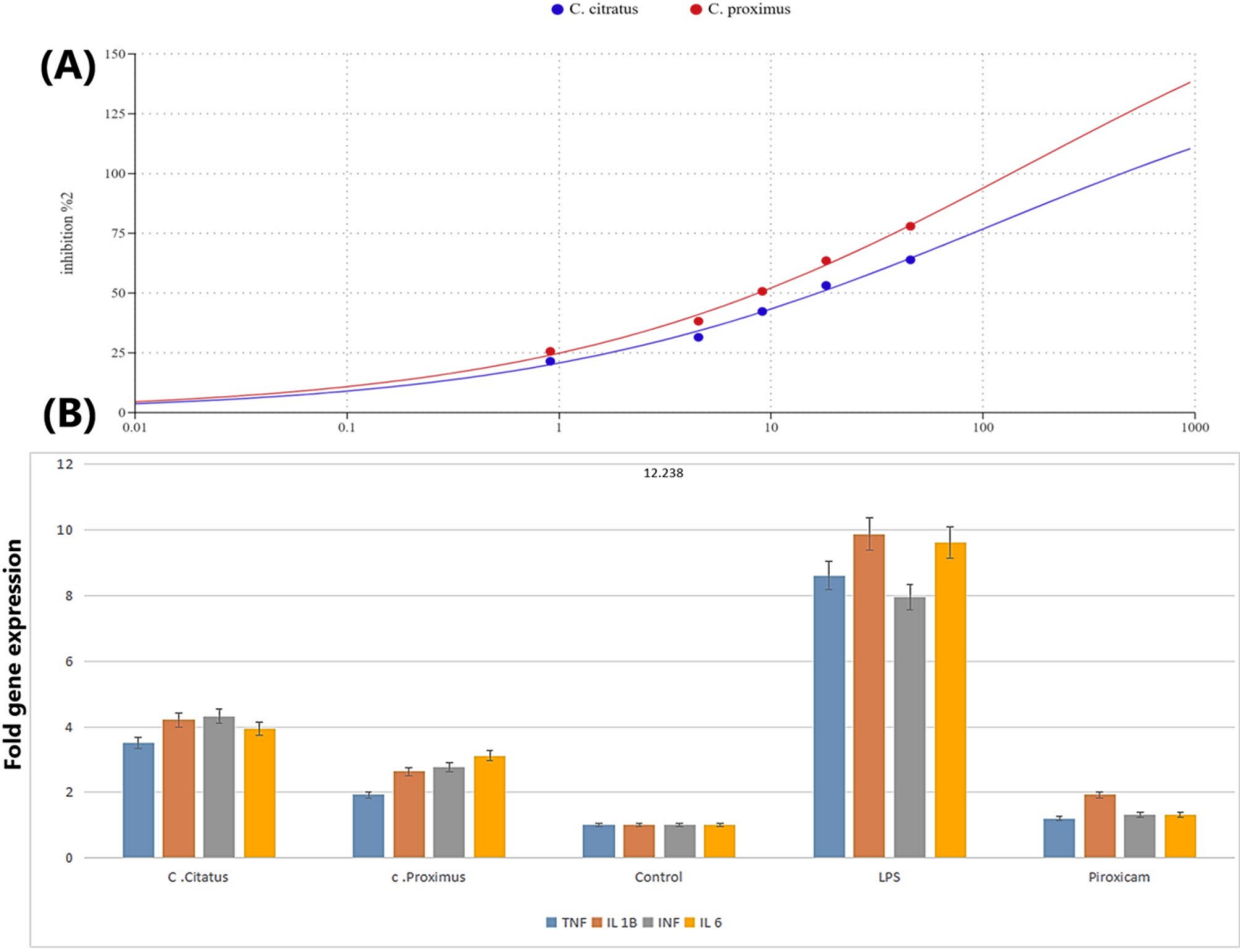
(**A**) Dose–Response Curve of Human Blood cells Membrane Stabilization. (B) The fold gene expression levels of four pro-inflammatory cytokines across different treatment conditions: *C. citratus, C. proximus*, control, LPS (lipopolysaccharide), and Piroxicam.

The anti-inflammatory potential of the plant essential oils was evaluated using the human red blood cell (HRBC) membrane stabilization assay. This model is biologically relevant because the HRBC membrane serves as an analog for the lysosomal membrane. In the inflammatory response, the rupture of lysosomal membranes leads to the extracellular release of hydrolytic enzymes and proteases, which induce tissue damage and exacerbate various inflammatory disorders. By inhibiting hypotonicity-induced membrane lysis, the essential oils demonstrate their ability to stabilize membrane integrity, hindering the inflammatory enzymes release, thereby exerting anti-inflammatory action. Based on the results, both essential oils exhibited significant membrane-stabilizing effects. Specifically, *C. proximus* demonstrated higher potency with an IC₅₀ of 8.94 μg/ml, compared to 14.65 μg/ml for *C. citratus* as presented in Fig. 3A. These findings suggest that both oils, particularly *C. proximus*, contain bioactive compounds capable of protecting lysosomal integrity and mitigating the inflammatory cascade.

#### 3.3.2. In vitro anti-inflammatory screening of different Cymbopogon species through inflammatory gene expression monitoring in LPS-stimulated white blood cells

First, the cytotoxicity of the essential oils on normal white blood cells using the MTT assay described by (Mosmann 1983). The EC100 values were determined to be 119.6 μg/ml for *C. proximus* oil, 144.4 μg/ml for *C. citratus* oil. These results indicate that the oils demonstrated distinct safety profiles in normal white blood cells.

The effective anti-inflammatory concentrations (EAICs) for each oil sample were measured against several inflammatory mediators: TNF-α, IL-1β, IFN-γ, and IL-6. For TNF-α, the EAICs were 50.54 μg/ml for *C. citratus*, 22.95 μg/ml for *C. proximus*, and 18.15 μg/ml for piroxicam. For IL-1β, the EAICs were 60.79, 31.44, and 28.95 μg/ml, respectively. In the case of IFN-γ, the values were 62.38, 33.24, and 19.8 μg/ml, while for IL-6, they were 57.04, 37.3, and 19.8 μg/ml, respectively. Consequently, *C. proximus* demonstrated superior anti-inflammatory activity compared to *C. citratus*, as indicated by lower EAIC values across all measured cytokines.

Figure 3B illustrates the fold gene expression levels of four pro-inflammatory cytokines TNF, IL-1β, INF, and IL-6 across five treatment conditions: *C. citratus*, *C. proximus*, a control group, LPS (lipopolysaccharide), and the anti-inflammatory drug Piroxicam. The study utilizes an in vitro model of inflammation to evaluate inflammatory gene expression. The control group demonstrated expression levels normalized to 1.0 for all cytokines. Conversely, LPS treatment—acting as the positive control—successfully induced a robust inflammatory state, dramatically increasing expression levels to 8.6 (TNF), 9.87 (IL-1β), 7.95 (INF), and 9.62 (IL-6).

Both *C. citratus* and *C. proximus* demonstrated notable modulatory effects on these LPS-induced cytokines. *C. citratus* showed moderate downregulation, reducing fold expression to 3.5 (TNF), 4.21 (IL-1β), 4.32 (INF), and 3.95 (IL-6). *C. proximus* displayed a more potent inhibitory effect than *C. citratus*, with values reaching 1.92 (TNF), 2.63 (IL-1β), 2.78 (INF), and 3.12 (IL-6).

This may suggest that *C. proximus* has a more potent anti-inflammatory profile, which could be valuable for therapeutic purposes, especially when compared to Piroxicam, which results in fold expressions of 1.21 (TNF), 1.93 (IL-1β), 1.32 (INF), and 1.32 (IL-6).

### In vivo* evaluation of the wound healing potential of C. proximus in a mouse excision model*

#### Histological evaluation of wound healing progress: evaluating the efficacy of C. proximus essential oil in a mouse excisional model

**Fig. 4:**
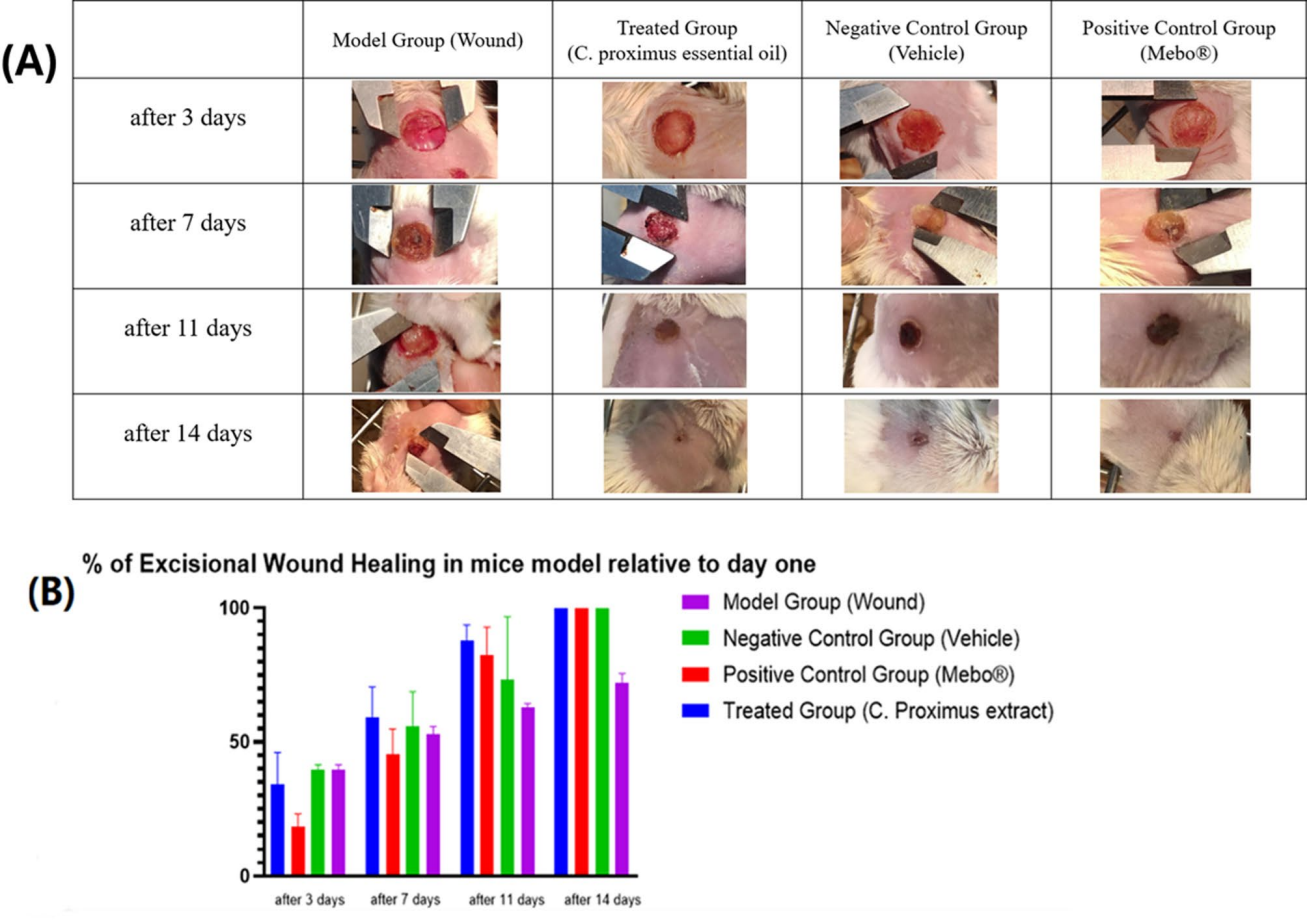
(**A**) A group of images illustrating the progression of wound healing in the different treatment groups. (**B**) The percentage of excisional wound healing progress in a Wistar rat model was evaluated over a 14-day period relative to the wound size on Day 1.

As shown in Fig. 4A and B, four treatment groups were compared: treated group with *C. proximus* essential oil 10% v/w, positive control group (Mebo®), negative control group (Vehicle), and model Group (Wound). At three days post-injury, the group treated with *C. proximus* essential oil demonstrated a wound healing rate of 34.22%. In contrast, the positive control group (Mebo®) showed a lower closure rate of 18.58%. Notably, the untreated wound model and the vehicle control groups exhibited higher initial healing percentages of 39.52% and 39.53%, respectively, during this early interval. By Day 7, all experimental groups demonstrated a progressive increase in wound closure. The *C. proximus* essential oil group maintained the highest healing rate at 58.99%. This was followed by the negative control group (Vehicle) (55.75%), the untreated model group (52.77%), and the Mebo® positive control group (45.43%). A significant advancement in wound closure was observed by Day 11, where both the *C. proximus* and Mebo® treatments reached approximately 87.98% and 82.59%, respectively, outperforming the Vehicle (73.16%) and the model (62.83%) groups. By the final assessment on Day 14, the *C. proximus*, Mebo®, and Vehicle groups achieved near-complete healing (approaching 100%), while the model group’s healing percentage was approximately 71.97%. Overall, the results indicate that *C. proximus* and Mebo® treatments promote more effective wound healing, resulting in near-complete closure by Day 14.

**Fig. 5:**
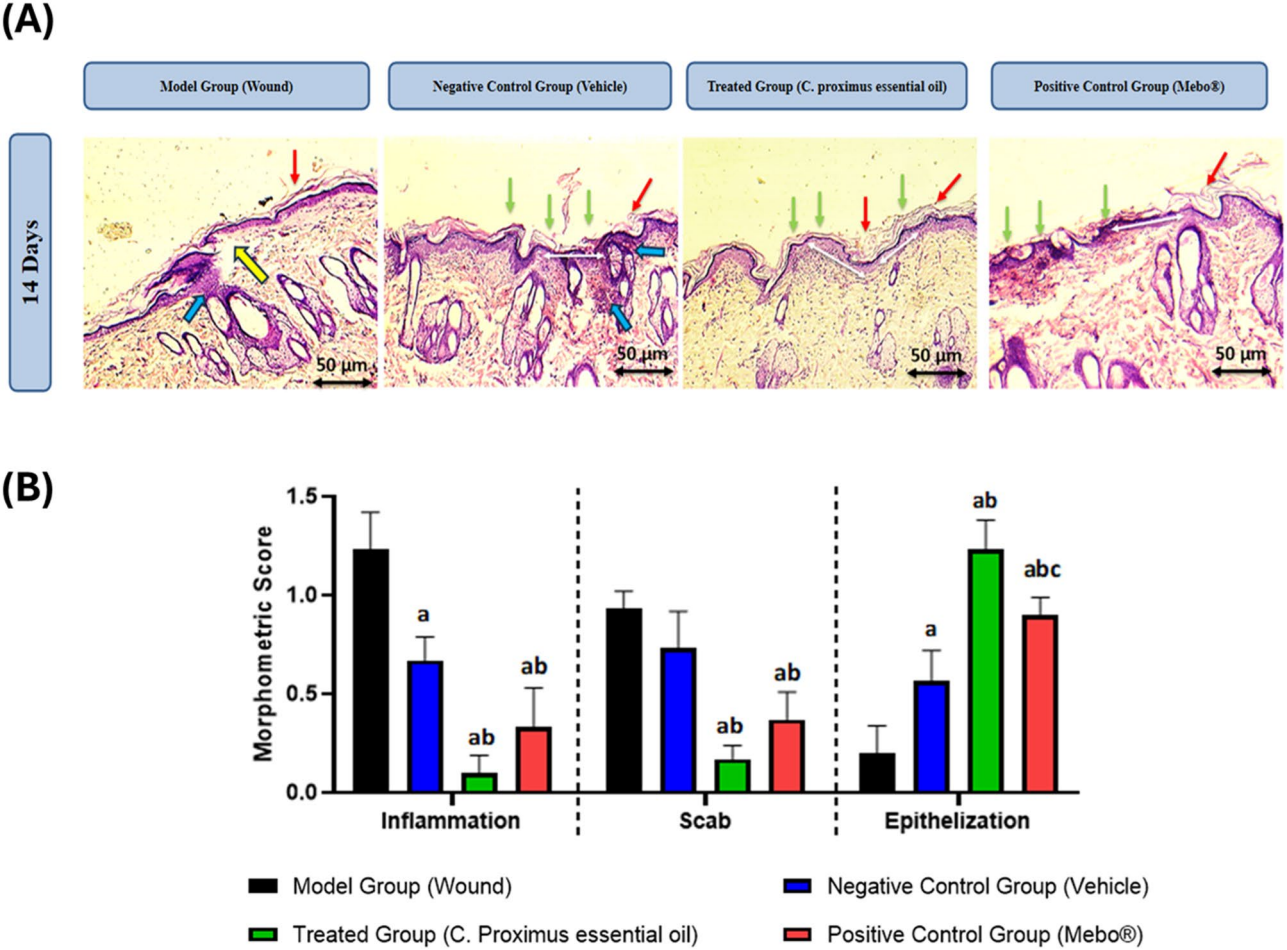
(**A**) Photomicrographs of HE-stained histological sections representative of the healing wound area in Wistar rats from different groups after 14 days of experiment (100 × , Scale bars: 50 μm). (**B**) Morphometric analysis of wound healing parameters inflammation, scab formation, and epithelialization in different treatment groups on Day 14 post-wound induction mice.

In Fig. 5A, full re-epithelialization of the wound surface is observed (green arrows), as well as the diameter of the healing area considerably (thin double-headed white arrows). Higher magnifications show thin keratinized squamous epithelium (red arrows). Incomplete closure of wound represented as yellow arrows. The fibrous connective tissue (TCF) shows persistence of chronic inflammatory infiltrate (light blue arrows) in negative control and Vehicle-treated groups, whereas inflammation was inconspicuous in the other groups.

On day 14, except for model Group (Wound) all other treated group presented full re-epithelialized wound surface and, although the reepithelization was quite advanced in *C. Proximus* treated group, a short non-epithelized area was seen. Both model Group (Wound) and negative control group (vehicle) exhibited mild chronic inflammatory infiltrate. All groups showed thin keratinized squamous epithelium of which *C. Proximus* treated group was the most adequate one. The fibrous connective tissue (TCF) shows persistence of chronic inflammatory infiltrate (yellow arrows) in model and vehicle treated groups, whereas inflammation was inconspicuous in the other groups.

Morphometric analysis of wound healing parameters inflammation, scab formation, and epithelialization in different treatment groups on Day 14 post-wound induction mice.

Data are expressed as Mean ± S.D. Statistical analysis was performed using one-way ANOVA in GraphPad Prism 8 software to determine the significance of differences among the groups. For each parameter, the data analysis was performed using the mean of the scores from each six sections (*n* = 6) analysed as shown in (Fig. 5B), (a) denotes that significant difference *p* < 0.05 vs. model Group (Wound), (b) denotes that significant difference *p* < 0.05 vs. negative control group (Vehicle), (c) denotes that significant difference *p* < 0.05 vs. treated group (*C. proximus* essential oil)**.**

Inflammation: which occurs at the beginning of the healing process with plasma extravasation, platelet aggregation, clot formation and recruitment of inflammatory (polymorphonuclear and mononuclear) cells to the wound site. The results indicate that all treatment groups significantly reduced the inflammation compared to the model Group (Wound) (1.23 ± 0.19). The treated group (*C. proximus* essential oil) demonstrated the greatest anti-inflammatory effect, recording the lowest mean score (0.10 ± 0.09), followed by the positive control group (Mebo®) (0.33 ± 0.20), and the negative control group (Vehicle) (0.67 ± 0.12). Statistical analysis, as indicated by the distinct letter notations ('a' and ‘ab’) on the bar chart, revealed a significant difference between the treatment groups and the model Group (denoted by 'a' above bars). The chart demonstrates that both the *C. proximus* and Mebo® treatment groups exhibited a significant difference compared with the vehicle group (denoted by 'b' above bars). However, no significant difference was observed between the *C. proximus* and Mebo® treatment groups (denoted by ‘ab’ above bars).

Concerning Scab formation which formed from cellular debris, fibrin, and red blood cells; remain as the epithelial tongue fills the injured site with epithelium. Scab formation, a high scab score was observed in the model Group (Wound) (0.93 ± 0.09). Both active treatments, *C. proximus* and Mebo®, significantly reduced the scab score compared to the model Group, suggesting an acceleration in the maturation and shedding of the necrotic tissue (denoted by 'a' above bars). The *C. proximus* essential oil group exhibited the lowest score (0.17 ± 0.17), indicating high efficacy in promoting wound surface clearance, followed by Mebo® (0.37 ± 0.14). This contrasts sharply with the vehicle group score (0.73 ± 0.19). Statistical analysis, as illustrated in the chart, indicates that both the *C. proximus* and Mebo® treatment groups exhibited a significant difference compared with the vehicle group (denoted by 'b' above bars). No significant difference was observed between the *C. proximus* essential oil and the Mebo®-treated groups (denoted by ‘ab’ above bar).

In the same context, Epithelialization of excisional wounds is a critical measure of tissue repair, in which epithelial cells depart from the edges of the injured epithelium towards the centre of the wound until they are joined and cover the whole site of the epithelium^4,27^. In the running study, epithelization showed the most pronounced differences. The negative control model Group (Wound) displayed the poorest epithelial repair (0.2 ± 0.14). In stark contrast, the *C. proximus* essential oil achieved the highest degree of re-epithelization (1.23 ± 0.15). The Mebo® group also demonstrated strong performance (0.97 ± 0.22), significantly higher than the vehicle group (0.57 ± 0.15). Statistical analysis, as illustrated in the chart, revealed that both the *C. proximus* and Mebo® treatment groups exhibited a significant difference compared with the vehicle group (denoted by 'b' above bars). Furthermore, a significant difference was observed between the *C. proximus* and Mebo® treatment groups (denoted by 'c' above bar).

These findings demonstrate that the *C. proximus* essential oil treated group consistently outperformed the model, vehicle, and Mebo® groups across all evaluated parameters, establishing its superior efficacy in modulating the inflammatory phase, clearing the scab, and accelerating the process of epithelization during wound healing.

#### Analysis of inflammatory biomarkers (IL-2, TGF-β1, and TNF-α) associated with wound healing tissues on Day 14 post-wound induction

**Fig.6:**
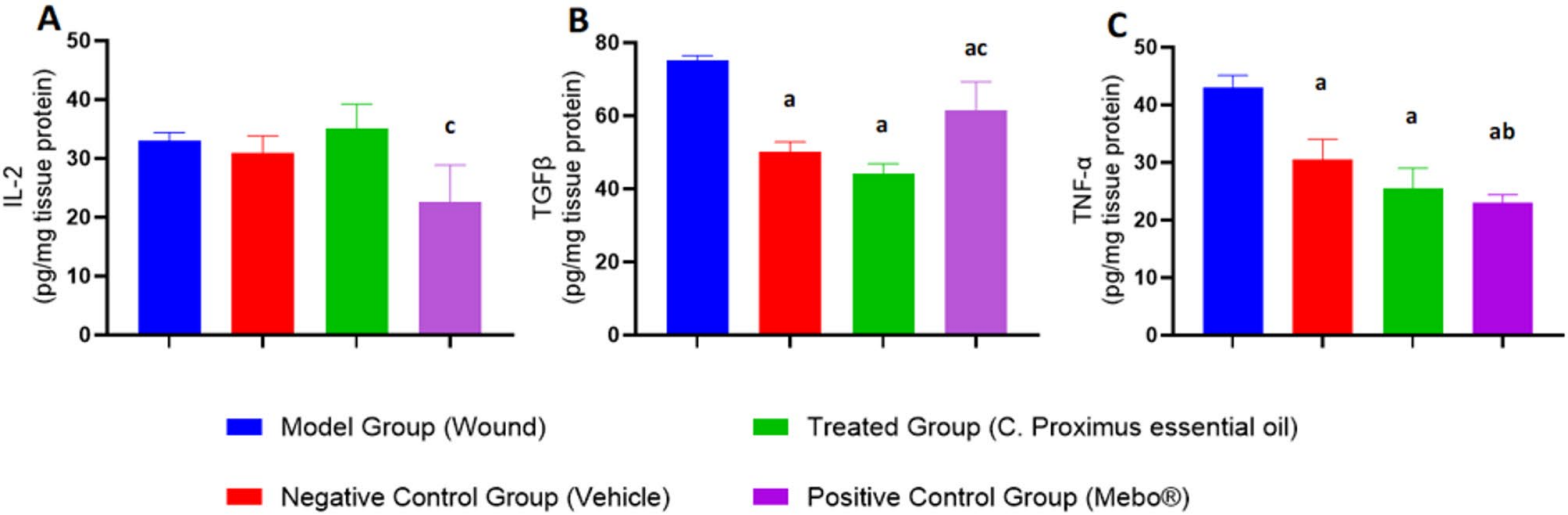
Biochemical parameters associated with wound healing process in different treatment groups on Day 14 post-wound induction mice.

Biochemical parameters associated with wound healing process in different treatment groups on Day 14 post-wound induction mice, where (A) represents IL-2 protein expression pg/mg tissue protein), (B) represents TGFβ protein expression pg/mg tissue protein) and (C) represents TNF-ꭤ protein expression pg/mg tissue protein) Data expressed as Mean ± S.D. Statistical analysis was performed using one-way ANOVA in GraphPad Prism 8 software to determine the significance of differences among the groups of (*n* = 6 mice) analysed as shown in (Fig. 6). (a) denotes that significant difference *p* < 0.05 vs. model Group, (b) denotes that significant difference *p* < 0.05 vs. Vehicle, (c) denotes that significant difference *p* < 0.05 vs. *C. Proximus* treated group.

In Fig. 6A**,** the model Group (Wound) demonstrated a high level of Interleukin-2 (IL-2) expression, with a mean concentration of (33 ± 1.14). The vehicle-treated group exhibited an increase in IL-2 expression, with a mean concentration of (31 ± 2.83). The *C. proximus* -treated group showed mean IL-2 concentration of (35 ± 4.24). Although this value was numerically higher than that of the vehicle group, the bar graph indicates that the difference was not statistically significant. This finding suggests that *C. proximus* exerted minimal or no additional stimulatory effect on IL-2 expression beyond that induced by the vehicle. Conversely, the Mebo®-treated group exhibited a mean IL-2 concentration of (22.5 ± 6.36), which was lower than both the vehicle and *C. proximus* groups. The statistical notation ('c') above the bar denotes a significant difference from the *C. proximus* group, indicating that Mebo® produced a comparatively reduced pro-inflammatory response.

In Fig. 6B, TGF-β1, as a growth factor, is a known proponent of dermal fibrosis by modulating infiltrated immune cells and the extracellular matrix^33^. The model Group (Wound) demonstrated a high level of Transforming Growth Factor Beta 1 (TGF-β1) expression, with a mean concentration of (75 ± 1.41). In contrast, the vehicle-treated group exhibited an increase in TGF-β1 expression, with a mean concentration of (50 ± 2.83). The *C. proximus* -treated group showed lower mean TGF-β1 concentration of (44 ± 2.83). Although this value was numerically lower than that of the vehicle group, the bar graph indicates that the difference was not statistically significant (denoted by 'a' above the bars). This finding also suggests that *C. proximus* exerted minimal or no additional stimulatory effect on TGF-β1 expression beyond that induced by the vehicle. Conversely, the Mebo®-treated group exhibited a mean TGF-β1 concentration of (61.5 ± 7.78), which was higher than both the vehicle and *C. proximus* groups. The statistical notation (‘ac’) above the bar denotes a significant difference from the *C. proximus* group. Notably, the *C. proximus* group exhibited a significantly reduced expression of inflammatory marker compared to the Mebo®-treated and model groups.

In Fig. 6C, the model Group (Wound) demonstrated a high level of Tumor Necrosis Factor α (TNF-α) expression, with a mean concentration of (43 ± 2.12). The vehicle-treated group exhibited a decrease in TNF-α expression, with a mean concentration of (30.5 ± 3.54). The *C. proximus* -treated group showed a low mean TNF-α concentration, reaching (25.5 ± 3.54). The Mebo®-treated group exhibited a mean TNF-α concentration of (23 ± 1.41), which was lower than both the vehicle and *C. proximus* groups. The statistical notation ('a') above the bars indicates a significant difference between the treatment groups and the model, suggesting that these groups exhibited a comparatively similar decrease in TNF-α expression. In contrast, the Mebo®-treated group demonstrated a significant difference from the vehicle group, as denoted by ('b') above the bars.

These findings indicate that all treatment groups exhibited comparable effects on the inflammatory biomarkers compared to the model group.

### Mechanistic insights of C. proximus essential oil components against wound using network pharmacology analysis

Network pharmacology analysis was employed as an exploratory computational strategy to predict potential molecular targets and signalling pathways associated with the major volatile constituents of *C. proximus* essential oil. This approach does not establish direct biological causality but provides a systems-level framework for generating testable mechanistic hypotheses.

#### Prediction of the main wound genes associated with C. proximus essential oil components

**Fig. 7:**
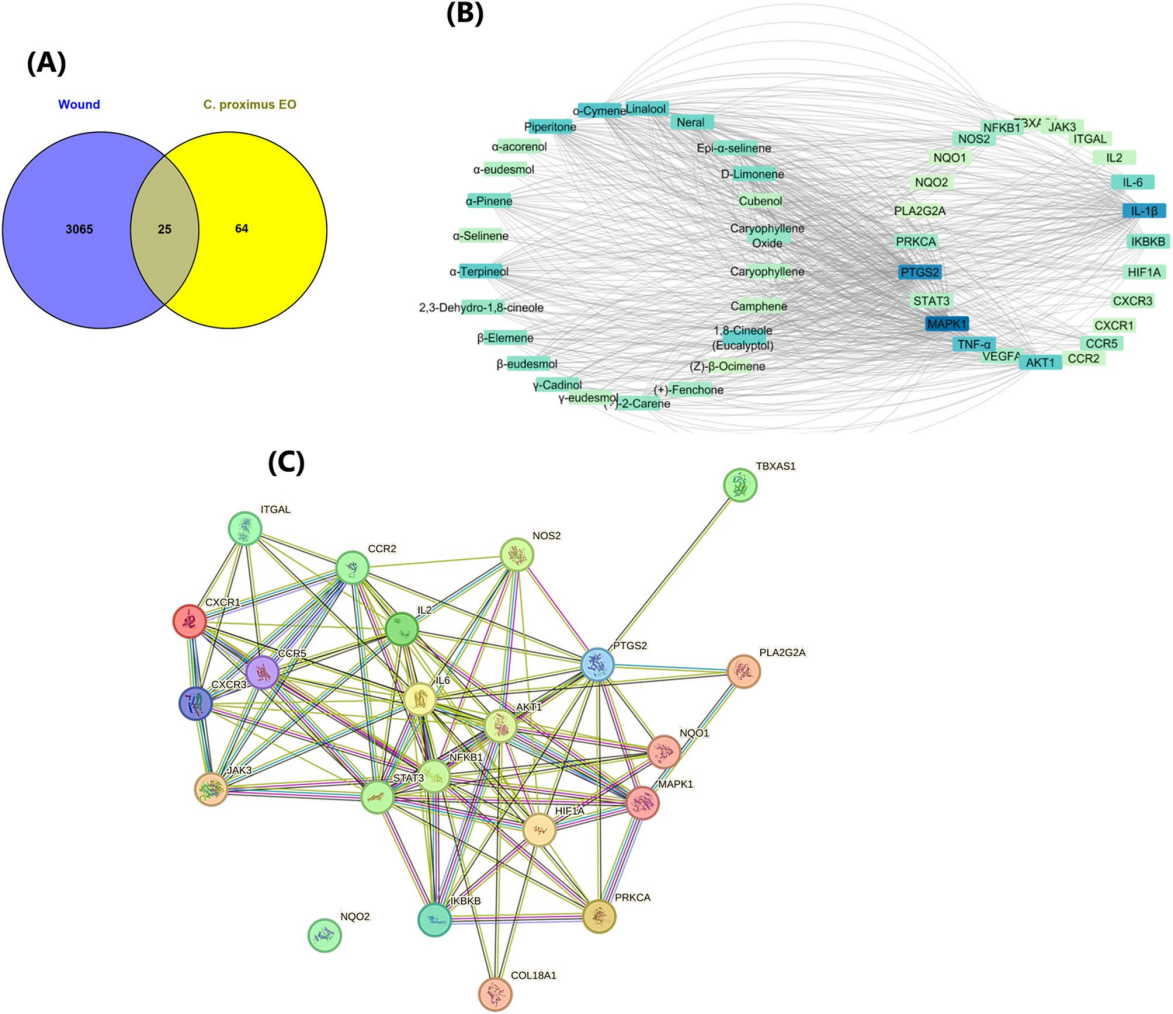
Network pharmacology analysis of *Cymbopogon proximus* essential oil in relation to wound healing. (**A**) Venn diagram showing the intersection between wound-related genes and predicted targets of *C. proximus* essential oil constituents, identifying 25 shared targets. (**B**) Compound–target interaction network illustrating the relationships between major volatile constituents of *C. proximus* essential oil and the intersecting wound-related targets; node size and color intensity reflect relative topological importance within the network. (**C**) Protein–protein interaction (PPI) network of the intersecting targets, highlighting highly connected hub proteins involved in inflammatory regulation, immune response, angiogenesis, and tissue remodeling. The networks collectively provide a systems-level, predictive view of potential molecular pathways through which *C. proximus* essential oil may contribute to wound-healing and anti-inflammatory effects.

In the current analysis, a total of 3065 genes were identified by profiling all genes linked to wound using genomic databases primarily, GeneCards and the Therapeutic Target Database (TTD). Meanwhile, 24 designated compounds from the *C. proximus* essential oil were submitted to the Swiss Target Prediction and STITCH 5.0 databases to predict their target genes. After eliminating unrelated genes using a Venn mapping program, 25 relevant targets were intersected with wound and *C. proximus* essential oil compounds (Fig. 7A). Then, compound-gene network was created to visually explore the functional interactions between chemical compounds and gene targets. As presented in (Fig. 7B), compound-gene network revealed 49 nodes and 473 edges, with 24 components and 25 potential targets related to wound where each compound was directly acted by an average of 3.717 linked targets, indicating multifaceted drug-target interactions. Of note, Uniprot database provides referenced data regarding the gene names of "*Homo sapiens*" species as listed in Table S1.

In accordance with the topological parameters summarized in Table 4, MAPK 1, PTGS2, IL-1β, TNF-α, AKT1, IL-6, IKBKB, and VEGFA were found to be the top-ranked putative genes highly correlated with the wound healing mechanism of *C. proximus* essential oil compounds as shown in Fig. 6B**.** While the relevant expected agents directly acting on these hub targets and might be the possibly active compounds synergistically function in wound mitigation included O-cymene, piperitone, α-terpineol, neral (β-citral or *cis*-citral), 1,8-cineole (eucalyptol), α-pinene, and D-limonene.

**Table 4:** The topological parameters for target gene nodes of *C. proximus* essential oil components.

Gene name	Betweenness Centrality	Closeness Centrality	Degree	Neighborhood Connectivity	Topological Coefficient
MAPK1	0.054863	0.401639	90	5.777778	0.318519
PTGS2	0.289815	0.521277	72	4.777778	0.188889
IL-beta	0.069552	0.415254	65	5.875	0.256579
TNF-alpha	0.068761	0.401639	45	5.7	0.313333
AKT1	0.018087	0.371212	40	5.8	0.342857
IL-6	0.001826	0.322368	30	6	0.555556
IKBKB	0.003251	0.322368	22	6.5	0.55
NOS2	0.22615	0.5	15	5.066667	0.193651
PRKCA	0.00673	0.340278	15	7.333333	0.487179
CCR5	0.026138	0.365672	14	5.125	0.4125
NFKB1	0	0.284884	13	6	0
HIF1A	0.058028	0.35	12	3.833333	0.283333
VEGFA	0.036289	0.382813	12	6	0.333333
STAT3	0.002549	0.326667	8	6	0.625
CXCR3	0.092041	0.360294	7	3.857143	0.285714
JAK3	0	0.30625	3	8	0
IL2	0	0.310127	3	9	0
CCR2	0	0.284884	2	6	0
ITGAL	0	0.310127	2	9	0
CXCR1	0	0.310127	2	9	0
NQO2	0	0.29878	1	6	0
TBXAS1	0	0.284884	1	6	0
NQO1	0	0.284884	1	5	0

In alignment with previous empirical literature findings^29,34^ Mitogen-activated protein kinases (MAPKs), including MAPK1, play crucial and diverse roles throughout the wound healing process, primarily by regulating cell proliferation and migration of keratinocytes and fibroblasts as well as extracellular matrix (ECM) remodeling, which are critical for wound closure (re-epithelialization)^35^. Essentially, PTGS2, also known as cyclooxygenase-2 (COX-2), which is focally produced in the wound area and peaks during the initial inflammatory phase, plays a vital role in wound healing by producing prostaglandins, primarily PGE2, which in turn regulates the inflammatory response and the recruitment of immune cells like macrophages, endothelial cells (angiogenesis), and epithelial cells (re-epithelialization)^36^. IL-1β, IL-6, and TNF-α are vital pro-inflammatory cytokines initiating wound healing by recruiting immune cells (neutrophils, macrophages) and activating endothelial cells for repair^37^. However, if overexpressed, they interfere with the wound healing process by fostering persistent inflammation, obstructing cell migration, and accelerating apoptosis^37^. Equally important, AKT1 (Protein Kinase B alpha) is crucial in different aspects of the wound healing process^38^. When activated, AKT1 promotes the growth and migration of keratinocytes and fibroblasts to accelerate wound closure as well as stimulates endothelial cell growth and nitric oxide (NO) production for angiogenesis promotion in the wound bed^39^. IKBKB plays a significant modulatory role in wound healing by balancing between inflammatory and tissue remodeling phases^40^. For clarity, it mediates inflammatory cytokine release to stimulate immune cell migration and cell proliferation, promoting proper tissue remodeling in the wound surface^40^. VEGFA (Vascular Endothelial Growth Factor A) is a well-known protein in wound healing through encouraging angiogenesis and revascularizing the damaged tissue, coordinating the injured tissue repair^24,32,41^.

In essence, these proteins just discussed act as master regulators in the wound healing process, orchestrating skin repair by mediating inflammation, supporting vessel growth, and tissue regeneration. Therefore, directly acting on these genes is considered an emerging strategy for developing effective wound-healing therapies, particularly for chronic wounds (like diabetic foot ulcers) that struggle to heal on their own.

In accordance with the relevant scholarly discourse, essential oil components, primarily *O*-cymene and piperitone, have been well-acknowledged for exerting promising antimicrobial, anti-inflammatory, antioxidant, and tissue-regenerating properties^42^. For clarity, O-cymene, piperitone, and 1,8-cineole (eucalyptol) mainly contribute to wound management through preventing wound infections and shortening the inflammatory phase of wound healing, which are particularly critical to enhance the natural healing process^43^. 1,8-cineole (eucalyptol) showed promise in improving closure rates and tissue regeneration in a nasal septum perforation rat model via inhibiting inflammatory mediators and boosting angiogenesis^44^.

Equally important, compelling evidence revealed that α-terpineol significantly promotes wound healing of deep burns by suppressing inflammatory cytokines such as TNF-α and oxidative stress, boosting cell migration, and stimulating angiogenesis, leveraging tissue repair in the wound area^45^. Further, a recent leading study demonstrated the promotional effect of α-pinene in wound healing and tissue remodeling through inhibiting inflammatory mediators (like TNF-α and IL-1β) and stimulating collagen deposition, accelerating wound contraction and closure^46^. Another in vivo study uncovered the profound effect of D-limonene in accelerating diabetic wound healing by significantly decreasing inflammatory cells and mediators, boosting granulation, epithelialization, and angiogenesis which are crucial for skin repair^47^.

In the same context, the wound crucial target genes were submitted to STRING ver. 10.5 database to create protein–protein interactions (PPIs) network offering a comprehensive view the molecular interactions occurring among these proteins within a biological system, gaining insights into disease mechanisms. As presented in Fig. 7C, The PPI network, which consists of high-confidence protein interactions with an enrichment *p*-value < 1.0e-16, pinpointed 22 nodes that represent biologically relevant genes and 105 edges indicative of protein–protein interactions, with AKT1, IL-6, IKBKB, NFKB1, MAPK 1, PTGS2, and STAT3 prominently positioned within the PPI network with a high degree of connectivity, affirming their multifaceted contributions to the pathogenesis of wound.

#### Forecasting the main signalling pathways of wound genes

**Fig. 8:**
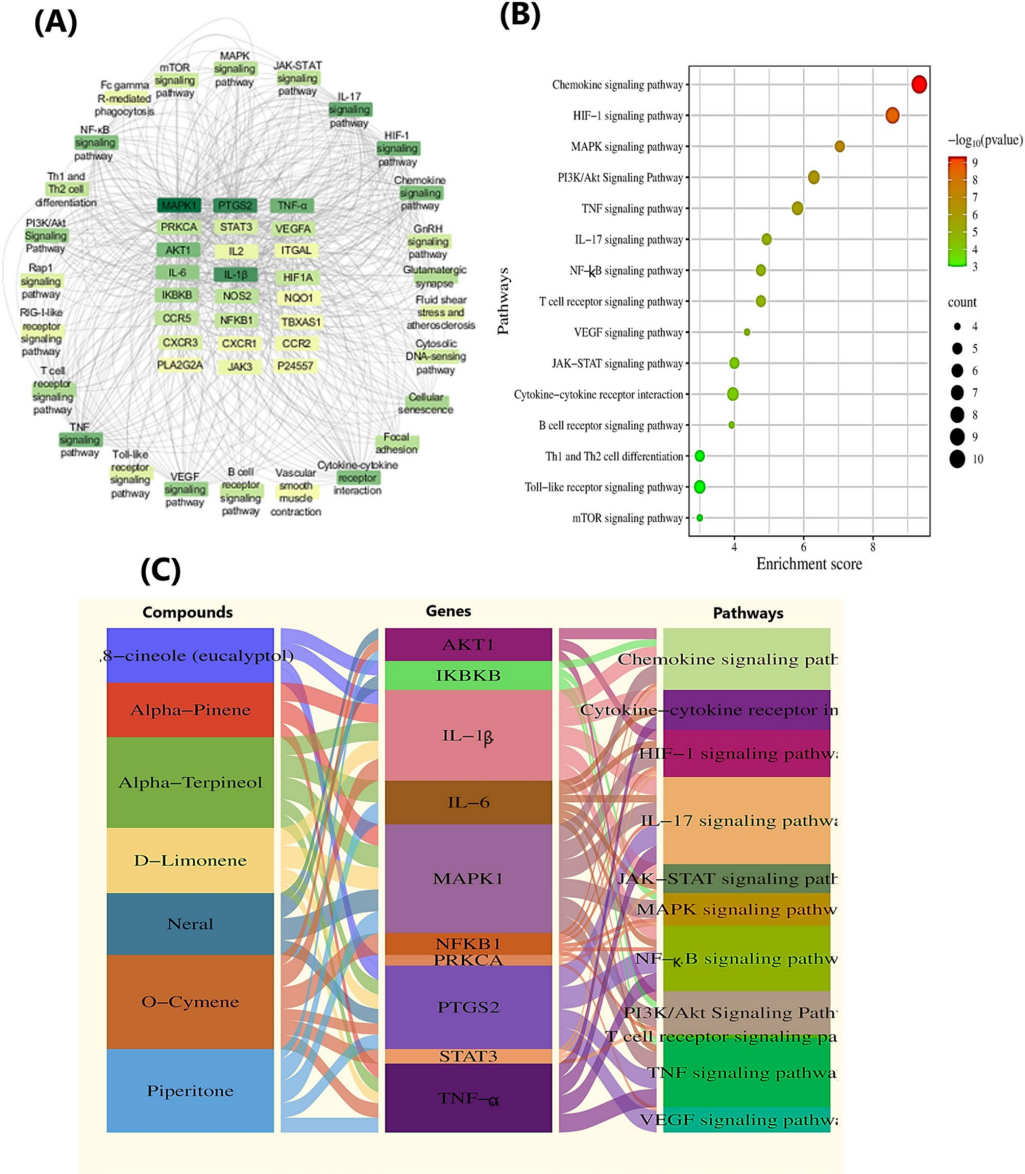
Pathway enrichment analysis and integrative compound–target–pathway mapping of *Cymbopogon proximus* essential oil. (**A**) Network visualization of enriched KEGG pathways^51^ associated with the intersecting wound-related targets, illustrating the relationships between key genes and multiple inflammation-, immune-, and wound-healing–related signaling pathways. (**B**) Bubble plot of KEGG pathway enrichment analysis showing the most significantly enriched pathways,bubble size represents the number of involved genes (count), while color intensity corresponds to − log₁₀ (p value). (**C**) Sankey diagram integrating major *C. proximus* essential oil constituents with their predicted target genes and the corresponding enriched signaling pathways, highlighting the multi-component, multi-target, and multi-pathway nature of the predicted biological actions. All pathway associations are derived from computational enrichment analysis and should be interpreted as hypothesis-generating rather than confirmatory.

To systematically elucidate the multi-waved molecular basis by which *C. proximus* essential oil components exert the wound healing effects, KEGG pathway analysis was conducted mapping the target genes with the cellular signaling pathways during skin wound. As depicted in Fig. 8A and Table S2 the highly relevant KEGG pathways to wound with false discovery rate < 0.05 were dissected. Among which, chemokine signalling pathway, MAPK signalling pathway, HIF-1 signaling pathway, TNF signaling pathway, NF- κB signaling pathway, PI3K-Akt signalling pathway, and IL-17 signalling pathway were the top- ranked pathways, co-regulated by the top-listed genes alluded above as illustrated in the bubble plot (Fig. 8B).

Parallel with literature^48^, chemokine signaling pathway is one of the notably activated cellular chemotaxis pathways in response to a wound, orchestrating the specific release of chemokines into the injury site to attract different immune cells (neutrophils, monocytes, and T-cells), which in turn trigger proliferation and tissue remodeling. Crucially, MAPK signaling pathway serves as a master regulatory pathway that manages the multi-stage process of wound healing from initial inflammation to the formation of new tissue via regulating the inflammatory phase along with triggering proliferation and migration of keratinocytes and fibroblasts into the wound area for proper tissue remodeling^35^. HIF-1 signaling pathway is a central regulator in different wound healing phases^49^. When activated, it promotes different cellular responses to the hypoxic environment at the injury site, mainly manifested by regulating infiltration of inflammatory cells like macrophages and neutrophils at the injury site to clear bacteria, angiogenesis, fibroblast proliferation, and collagen deposition for proper skin repair^49^. NF-κB signaling pathway plays a central role in wound healing, regulating initial inflammation and subsequent phases like proliferation, angiogenesis, and tissue remodeling when normally regulated but causing persistent inflammation and chronic issues if dysregulated^50^. Analogously, the normal activation of PI3K/Akt signaling pathway centrally modulates the key cellular functions during wound healing process such as initial inflammation, proliferation, migration, survival, angiogenesis which are essential for normal skin repair and homeostasis, while its dysregulation can contribute to chronic wounds and other skin disorders^38^. Both TNF and IL-17 signaling pathways are associated with immune regulation and inflammatory responses in wound healing^37^.

In the light of network findings, the selective targeting of the hub wound genes and the balanced activation of interconnected signaling pathways by *C. proximus* essential oil compounds facilitate a more coordinated and efficient progression through the complex stages of wound healing (hemostasis, inflammation, proliferation, and remodeling). To shed some light on the main multi-level interactions among the main biological entities, such as active compounds, target genes, and pathway, a sankey plot (Fig. 8C) was created, simply illustrating the main functional associations between different network components.

### Molecular docking analysis

**Fig. 9:**
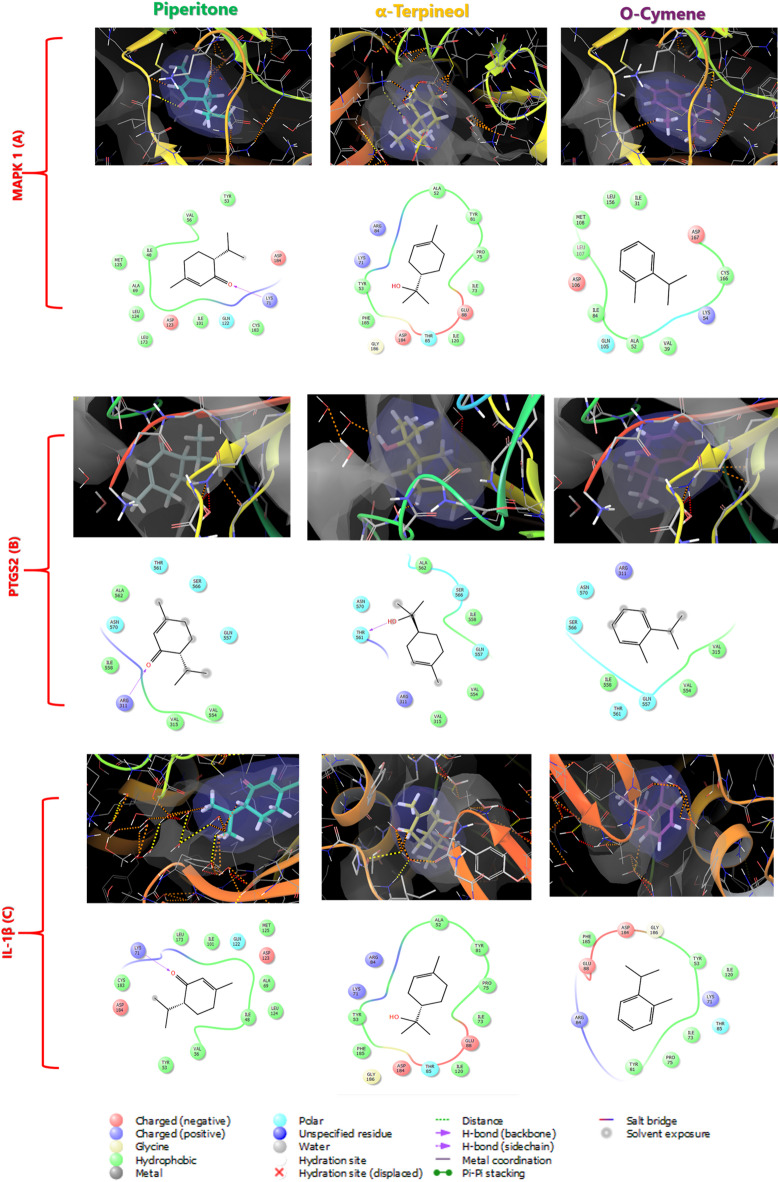
Molecular docking analysis of selected Cymbopogon proximus essential oil constituents with key inflammation-related targets. Representative three-dimensional binding poses (upper panels) and two-dimensional ligand–protein interaction maps (lower panels) illustrating the predicted interactions of piperitone, α-terpineol, and o-cymene with (**A**) MAPK1, (**B**) PTGS2 (COX-2), and (**C**) IL-1β.

In accordance with network pharmacology findings, molecular docking analysis was performed to demonstrate the binding orientations (pose) between the top efficacy compounds in *C. proximus* essential oil (O-cymene, piperitone, and α-terpineol) and the top-ranked genes namely, MAPK 1, PTGS2, and IL-1β. The gathered data revealed that the candidate compounds demonstrated strong binding affinities for the target protein with promising docking scores, assuring good stability and appropriate geometric fitting within the protein catalytic pockets. More specifically, both α-terpineol and O-cymene were found to bind effectively to the active site of the MAPK 1 protein with high affinity (XP Gscore = −6.43 kcal/mol and −6.04 kcal/mol, respectively) primarily through electrostatic interactions with ASP 184, ASP 106, ASP 167 and GLU 88 as well as hydrophobic interactions with TYR 81, TYR 53, PHE 185, PRO 75, MET 108, ILE 31, and ILE 73 as presented in Fig. 9A. Also, piperitone demonstrated stable fitting within the MAPK 1 binding pocket, via forming hydrogen bonds with LYS 71 complemented by significant van der Waals interactions with VAL 156, LEU 124, ALA 69, CYS 183, and ILE 102. Importantly, the 2D and 3D diagrams in Fig. 9B illustrate that piperitone exhibits a favorable binding tendency toward the PTGS2 catalytic site, with a good binding score of -5.46 kcal/mol, where the compound’s carbonyl group engaged in hydrogen bonds interactions with the residue MET 92 and its p-menthane skeleton was stabilized within the pocket via polar and hydrophobic interactions with several surrounding residues, specifically ASN 570, GLN 557, SER 566, THR 561, ALA 562, ILE 558, VAL 315, and VAL 554. Similarly, α-terpineol bound favorably to the PTGS2 catalytic pocket, where the complex was stabilized by hydrogen bonds between the compound’s hydroxyl group and THR 561 along with polar and hydrophobic interactions with GLN 557, SER 566, ALA 562, ILE 558, VAL 315, and VAL 554 residues. O-cymene, piperitone, and α-terpineol exhibited comparable and favorable binding affinities for the IL-1β protein catalytic site, registering favorable Gscores of −6.85, −6.46, and −5.77 kcal/mol via forming electrostatic and hydrophobic interactions with the amino acid residues within catalytic pocket (Fig. 9C). These interactions collectively orient O-cymene, piperitone, and α-terpineol optimally within the IL-1β binding site and stabilizing the resulting complexes.

## Conclusion

The present study provides experimental support for the traditional use of *Cymbopogon proximus* in the management of inflammatory conditions and wound-related disorders. Through integrated chemical characterization and biological evaluation, the essential oil of *C. proximus* was shown to exhibit anti-inflammatory activity and to promote wound healing under the applied experimental conditions. The observed biological effects were most consistently associated with modulation of TNF-α and with improvements in histological and wound closure parameters, while other cytokine responses, including IL-2 and TGF-β1, were modest or variable.

Comparative evaluation with a reference topical formulation indicated that the wound-healing and anti-inflammatory effects of *C. proximus* essential oil were generally comparable rather than uniformly superior. These findings suggest that the essential oil may act as a supportive or complementary agent in wound management, rather than as a replacement for established therapies. Chemical profiling revealed a piperitone-dominant composition, highlighting the potential contribution of monoterpene constituents to the observed activities, although causal relationships remain to be experimentally confirmed.

Exploratory network pharmacology and molecular docking analyses offered predictive insights into possible molecular targets and pathways related to inflammation and tissue repair; however, these in silico findings should be regarded as hypothesis-generating and require further validation using target-specific and mechanistic assays. In addition, limitations related to single-location sampling, absence of seasonal chemotype assessment, relative (rather than absolute) quantification of volatile constituents, and restricted dose–response evaluation should be acknowledged.

Overall, this work contributes to the growing body of evidence-based ethnopharmacology by bridging documented traditional knowledge with experimental pharmacological validation. Future studies should focus on expanded toxicological evaluation, dose optimization, chemotype variability, and mechanistic confirmation to further define the therapeutic relevance and translational potential of *C. proximus* essential oil in inflammatory and wound-healing contexts.

## Data Availability

Data will be available upon request.
